# Optimising Retraining Frequency for a Paediatric Emergency Department Admission Prediction Model: Development and Temporal Validation Using Real‐World Data

**DOI:** 10.1111/1742-6723.70271

**Published:** 2026-05-06

**Authors:** Ethan Williams, Toshi Sinha, Mark Lyttle, Yogesan Kanagasingam

**Affiliations:** ^1^ Perth Children's Hospital Emergency Department Nedlands Australia; ^2^ The University of Notre Dame Australia, School of Medicine Fremantle Australia

**Keywords:** admission prediction, artificial intelligence, concept drift, machine learning, paediatric emergency medicine

## Abstract

**Objective:**

To analyse temporal performance drift and optimal retraining frequency for an ensemble machine learning model to predict inpatient admission from paediatric emergency department (ED) triage data.

**Methods:**

This study utilised 409,307 ED presentations from 1 July 2018 to 30 June 2024 at Perth Children's Hospital. An ensemble stacking model (XGBoost, TabNet, multi‐layer perceptron and logistic regression base learners with a logistic regression meta‐learner) incorporated structured triage features and tuned BioClinicalBERT‐derived embeddings from free‐text notes. The model ran prospectively through a 5‐year rolling‐window simulation, testing nine retraining cadences from weekly to triennial and a static model. Training, retraining and validation datasets were temporally separate and prior to the test set. Primary outcomes were discrimination via the area under the receiver operator characteristic (AUROC) and calibration as absolute mean daily bed error (AMDBE).

**Results:**

Weekly retraining achieved a mean AUROC of 0.843 (SD 0.016) and AMDBE of 2.57 (SD 1.79) over the 5‐year simulation. Fortnightly and monthly cadences were non‐inferior (AMDBE 2.61 and 2.73), whereas longer intervals showed progressive calibration degradation (*p* < 0.001) and stable AUROC. Concept drift was most pronounced in the static model, with a mean AMDBE of 10.6 in 2024 compared to 1.79 for the weekly model. Notably, monthly retraining required only 25% of the weekly computational burden with non‐inferior performance.

**Conclusion:**

Monthly, or more frequent, model retraining sustains discrimination and calibration for paediatric ED admission prediction. This effectively mitigated concept drift and enabled accurate simulated daily bed‐demand forecasting, providing evidence to support the clinical testing of such modelling.

## 1 Introduction

Emergency departments (ED) globally are under escalating pressure from rising patient volumes and disease complexity, negatively impacting the quality, safety and efficiency of patient care [1, 2]. Delays to care in ED are associated with longer inpatient stays and increased 30‐day mortality [3]. There is growing interest in tools that can identify (before full clinician assessment) which patients require hospital admission, as accurate prediction‐based capacity planning optimises bed and resource allocation.

Numerous studies have explored models, including machine learning (ML), which predict inpatient admission at the time of ED triage, the earliest point in a patient's hospital journey [4, 5, 6]. Variations of decision trees and neural networks are increasingly used to process complex nonlinear relationships in ED data to predict admissions [4, 5, 7, 8]. Increasingly, natural language processing (NLP) of unstructured data from free‐text triage notes is used to improve model performance [9, 10]. Many models with sound methodology have often only trained and tested on adult or mixed populations, whilst paediatric EDs face unique challenges [4, 5, 11, 12]. Their presenting concerns differ vastly from adult populations, and seasonal demand patterns are highly variable and may contribute to increased covariate drift [11, 12]. Within the paediatric ED setting, incorporating NLP techniques into deep learning models has also improved performance [5, 10]. However, many models are retrospective, reporting pooled results without temporal validation, retraining or consideration of concept drift [4, 5, 13]. Concept drift is the temporal degradation of model performance as clinical populations, workflows and data distributions evolve over time. Concept drift must be understood and addressed prior to real‐world implementation, to ensure safety and maximise clinical utility, especially in paediatric settings.

This study aimed to develop and temporally evaluate an ensemble ML model's performance in predicting hospital admission from routine triage features in a paediatric ED, using clinically relevant outcome measures. During this 5‐year simulation, we explored variable retraining frequencies to maintain optimal model discrimination and calibration over time.

## 2 Methods

### 2.1 Study Design and Overview

This in silico simulation study evaluated a ML ensemble's performance in predicting inpatient admissions from ED triage data at a tertiary paediatric centre. An ensemble stacking model was created, with base learners including mandatory XGBoost (XGB) and optional multilayer perceptron (MLP), TabNet and logistic regression (LR), with a LR meta‐learner to blend the ensemble. Simulation then tested the model with 10 retraining cadences: weekly, fortnightly, monthly, bimonthly, trimonthly, half‐yearly, yearly, biennial, triennial and no retraining (referred to as the static model). The simulation ran prospectively through historic data, mimicking real‐world deployment, for temporal validation. An in‐depth description of model development including technical details and visualisation of the training process can be found in Appendix S1. The study strictly adhered to the Transparent Reporting of a Multivariable Prediction Model for Individual Prognosis or Diagnosis for Artificial Intelligence (TRIPOD+AI) guidelines for development and reporting, and the Prediction Model Risk of Bias Assessment Tool (PROBAST) for bias evaluation [13, 14]. These can be found in Appendix S2.

The study was approved by the Child and Adolescent Health Service (CAHS) Human Research Ethics Committee (RGS0000007381) and the University of Notre Dame Australia Human Research Ethics Committee (2025‐019). It was conducted on a secure platform, MERLIN, within the CAHS network. It comprises four NVIDIA H100 GPUs, 64 Intel(R) Xeon(R) Gold 6448Y CPUs and 512 Gb of RAM, and was approved for research purposes. The code, or sections of it, may be available to researchers upon reasonable request.

### 2.2 Data Source and Cohort

Deidentified data were sourced from Perth Children's Hospital, Western Australia's sole tertiary paediatric centre, serving approximately 2.6 to 3 million people during the study. The dataset spanned 1 July 2018, to 30 June 2024, including 409,784 ED presentations and 82,549 inpatient admissions from ED. The first year of data was used only for training and validation, whilst the subsequent 5 years were used for the rolling retraining, validation and testing window. All presentations were included to reflect real‐world heterogeneity. Exclusion criteria applied for incomplete arrival date (*n* = 0) or a non‐admission or non‐discharge outcome (*n* = 447), comprising overage patients sent to adult services at triage or patient death in ED (*n* < 50).

Features available at triage included demographics (age in years, sex, postcode), temporal data (arrival time and date), triage priority (Australasian Triage Scale), chief complaint and code, historical presentations (days since last presentation and admission, number of presentations and admissions in prior 12 months), derived temporal features (sine/cosine day cycles, weekend indicator, shift bins: early morning [0–8 h], morning [8–14 h], afternoon [14–18 h], evening [18–24 h]) and optional NLP embeddings from two triage nurse free‐text fields. Vital signs are not routinely collected at triage in this centre, so were not available for this study. The binary outcome was inpatient admission from ED, either to an inpatient ward or emergency short stay, versus discharge home, including discharge against medical advice. This was chosen to best represent the bed demand on the hospital. Missing categorical features were replaced using a fallback label ‘unknown’. Age, sex, triage score and text fields had no missing values. When the field was empty for days since last admission or presentation, the value was imputed as patient's age in days. Other numeric features were imputed as 0, with postcode having the largest number of missing values (*n* = 3093).

### 2.3 Evaluation Metrics and Statistical Analysis

The primary outcome measures were area under the receiver operating characteristic curve (AUROC) for discrimination and absolute mean daily bed error (AMDBE) for clinical utility and calibration. AMDBE is computed as the daily mean absolute difference between daily patients' summed probabilities of admission and actual number of admissions that day. For example, if there are three patients in the ED where the model predicts the probability of admission is 0.1, 0.4 and 0.7 respectively, the model is predicting a bed requirement of 1.2 for this cohort. Then, AMDBE across a series of days is calculated as the mean absolute bed error across these days. For example, if the model underpredicts admissions by 1.5 beds on Monday, 3.0 beds on Tuesday and 4.5 beds on Wednesday, and overpredicts by 3.0 beds on Thursday, the AMDBE across those 4 days is (1.5 + 3.0 + 4.5 + 3.0)/4 = 3.0 beds. The model intends to summate a daily prediction of required beds via pooled probabilities from ED triage data, rather than directly predict or influence a patients' disposition. As the focus was on bed prediction, socioeconomic and racial data were not collected and therefore not included in analysis to avoid bias. Additionally, we assessed covariate drift across the 5‐year testing window using domain classification, principal component analysis, reconstruction error and repeated‐period performance monitoring. The full description of drift analysis is provided in Appendix S1.

Secondary measures included accuracy, precision, sensitivity, specificity, negative predictive value, F1 score, Brier score, calibration slope and intercept, expected calibration error and SHAP values for global feature importance. Weekly primary outcome measures were collected and as the distribution was non‐parametric via Shapiro–Wilk testing, Wilcoxon signed‐rank test was performed with Bonferroni correction (*α* = 0.05/9). Plotting of the primary outcome over time to explore temporal drift was also completed to demonstrate the drift in performance over time.

## 3 Results

### 3.1 Dataset Characteristics

The dataset comprised 409,784 ED presentations over 6 years, with 477 cases removed due to being overage or death in the ED. Table 1 presents the demographics of the 409,307 included ED presentations and 82,549 admissions (20.2%). There was a slight male preponderance (55%); and admission rates were higher for presentations between midnight and 08:00 (25.3%), infants under 1 year (23.5%), regional and remote patients (47.6%) and triage priority 1 and 2 cases (91.0% and 56.3%, respectively). The average daily presentations were 187 (SD 30.9; range 81–304), with August being the busiest month (mean 214, SD 17) and January the least busy (mean 161, SD 10). Presentation variance exceeded the mean in all periods with an Index of Dispersion (ID) of 1.6 to 6.7, demonstrating substantial overdispersion relative to a Poisson assumption. Admission rates had less variability, with a mean of 37.7 (SD 3.3) and ID of 0.92 to 1.64, with Table 2 displaying the variability for all quarters.

**TABLE 1:** Study population demographics.

Category		Presentations	Admissions	Percentage admitted
Year	2018 (1 Jul— 31 Dec)	33,425	6491	19.4%
	2019	69,668	13,746	19.7%
	2020	62,313	12,252	19.7%
	2021	71,043	13,788	19.4%
	2022	68,759	14,158	20.6%
	2023	71,029	15,105	21.3%
	2024 (1 Jan—30 Jun)	33,547	7009	20.9%
Triage score	1	2632	2396	91.03%
	2	44,655	25,144	56.31%
	3	122,933	36,520	29.71%
	4	235,877	18,456	7.82%
	5	3687	33	0.90%
Sex	Female	184,428	36,348	19.71%
	Male	225,332	46,194	20.50%
	Other	24	7	29.17%
Age in years	< 1	63,559	14,935	23.50%
	1–4.9	145,096	28,608	19.70%
	5–9.9	94,217	17,194	18.20%
	10–14.9	87,277	17,113	19.60%
	15–17.9	19,455	4627	23.80%
	18+	180	72	40.00%
Postcode	Metropolitan	385,957	72,474	18.8%
	Regional or remote	20,734	9877	47.6%
	No postcode	3093	198	6.4%
Time of arrival (divided by shift categories)	0000–0800	44,397	11,254	25.3%
	0800–1400	134,679	24,495	18.2%
	1400–1800	100,448	19,761	19.7%
	1800–2400	130,260	27,039	20.8%
Emergency department bed allocation stream	Resus	2805	2467	87.95%
	Pods	302,976	73,769	24.35%
	Fast track	103,362	6229	6.03%
	Other	641	84	13.10%

**TABLE 2:** Variability in presentations and admissions and performance of the weekly retraining model.

Quarter	Presentations				Admissions				Mean AMDBE
	Mean	SD	Variance	ID	Mean	SD	Variance	ID	
2019 Q3	200.1	22	483.1	2.41	41.8	6.9	47.2	1.13	2.81
2019 Q4	185.8	19.1	363.3	1.96	36.9	6.3	39.8	1.08	2.13
2020 Q1	168.5	26.5	699.6	4.15	34.1	7.3	53	1.55	2.32
2020 Q2	128.3	24.5	597.8	4.66	27.7	6.7	45.3	1.64	2.03
2020 Q3	169.1	27.2	742	4.39	33.4	6.9	47.6	1.43	3.05
2020 Q4	210	27.4	749.1	3.57	38.8	6.5	42.3	1.09	3.44
2021 Q1	180.6	25.5	651.8	3.61	35.9	6	36	1	2.89
2021 Q2	182.4	17.2	294.1	1.61	36.6	5.8	33.6	0.92	2.04
2021 Q3	211.8	37.8	1428.1	6.74	43.4	6.5	42.3	0.97	5.01
2021 Q4	203.3	23.3	542.4	2.67	39.8	6.1	37.2	0.93	3.08
2022 Q1	164.9	16.7	278.6	1.69	33.1	5.8	33.6	1.02	2.15
2022 Q2	181.1	25.3	642.1	3.55	36.4	6.2	38.4	1.06	2.83
2022 Q3	207.6	25.1	628	3.02	42	6.9	47.6	1.13	2.55
2022 Q4	199.2	21.8	475.2	2.39	38.9	6	36	0.93	2.46
2023 Q1	182.6	21.6	465.3	2.55	36	5.9	34.8	0.97	2.64
2023 Q2	205.3	28.1	787.9	3.84	40.3	6.3	39.7	0.98	2.21
2023 Q3	208.3	21.4	458.8	2.2	41.9	6.4	41	0.98	2.56
2023 Q4	182.1	20.8	433.5	2.38	39.1	6	36	0.92	1.67
2024 Q1	173.2	19.8	392	2.26	35	5.8	33.6	0.96	2.25
2024 Q2	195.4	20.9	437.6	2.24	40.3	6.3	39.7	0.98	1.32

### 3.2 Model Performance Across Training Cadences

**FIGURE 1:**
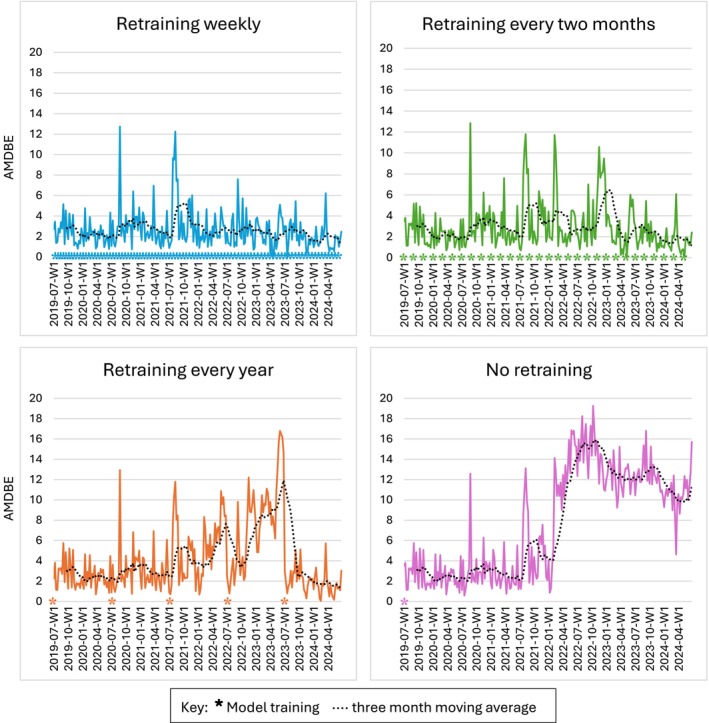
AMDBE over the 5‐year simulation for models of different retraining frequencies.

Weekly retraining yielded the best overall performance (AUROC 0.843, SD 0.016, 95% CI 0.806–0.867; AMDBE 2.57, SD 1.79, 95% CI 0.54–7.41), significantly outperforming (*p* < 0.001) all cadences in AMDBE other than fortnightly and monthly. Fortnightly (AUROC 0.843, AMDBE 2.61) and monthly (AUROC 0.844, AMDBE 2.73) retraining were not significantly inferior in AMDBE. The comparison between all cadences' performance is displayed in Table 3. Covariate drift was modest in early years but increased from January 2022 onwards, reflected in worse static model performance as visualised in Figure 1. Performance drift was more pronounced in calibration than discrimination metrics, as AUROC for the static model declined from 0.852 in 2019 to 0.799 in 2024, whereas AMDBE rose from 2.73 in 2019 to 10.6 in 2024. This increased AMDBE reflects progressive underprediction of admission at higher predicted‐risk levels after 2022, with further covariate and performance drift data available in Appendix S1. Weekly retraining mitigated concept drift by minimising discrimination drift with an AUROC of 0.851 in 2019 to 0.838 in 2024, and improving calibration as AMDBE dropped from 2.47 in 2019 to 1.78 in 2024.

**TABLE 3:** Primary outcome measure means for each model over the 5‐year simulation.

Cadence	Metric	Mean (SD)	Median (IQR)	*p* vs. weekly	Effect size	Effect label
Weekly	AUROC	0.843 (0.016)	0.845 (0.023)	N/A	N/A	N/A
	AMDBE	2.572 (1.793)	2.136 (1.685)	N/A	N/A	N/A
Fortnightly	AUROC	0.843 (0.015)	0.845 (0.021)	0.23	−0.103	Negligible
	AMDBE	2.606 (1.811)	2.172 (1.841)	0.52	0.056	Negligible
Monthly	AUROC	0.844 (0.015)	0.844 (0.021)	0.10	−0.131	Negligible
	AMDBE	2.727 (1.955)	2.147 (1.866)	0.34	−0.076	Negligible
Bimonthly	AUROC	0.841 (0.015)	0.843 (0.021)	0.079	0.136	Negligible
	AMDBE	2.880 (2.223)	2.147 (2.180)	< 0.01	−0.306	Small
Trimonthly	AUROC	0.842 (0.017)	0.844 (0.023)	0.39	0.065	Negligible
	AMDBE	3.690 (4.638)	2.476 (2.145)	< 0.01	−0.306	Small
Semiannual	AUROC	0.840 (0.020)	0.841 (0.029)	0.042	0.153	Negligible
	AMDBE	3.527 (2.976)	2.554 (2.979)	< 0.01	−0.402	Small
Annual	AUROC	0.837 (0.020)	0.839 (0.029)	< 0.01	0.431	Small
	AMDBE	4.076 (3.373)	2.963 (3.553)	< 0.01	−0.559	Medium
Biennial	AUROC	0.831 (0.022)	0.832 (0.034)	< 0.01	0.688	Medium
	AMDBE	3.939 (2.786)	2.821 (4.133)	< 0.01	−0.505	Medium
Triennial	AUROC	0.843 (0.017)	0.845 (0.021)	0.953	−0.004	Negligible
	AMDBE	5.855 (3.939)	4.504 (6.784)	< 0.01	−0.811	Large
Static	AUROC	0.822 (0.026)	0.824 (0.039)	< 0.01	0.843	Large
	AMDBE	7.691 (5.271)	8.058 (9.956)	< 0.01	−0.823	Large

Secondary metrics for the weekly model included mean accuracy 0.846 (SD 0.011), sensitivity 0.452 (SD 0.045), specificity 0.947 (SD 0.011), precision 0.686 (SD 0.045), NPV 0.871 (SD 0.014), F1 0.543 (SD 0.037), Brier 0.113 (SD 0.007), calibration slope 1.041 (SD 0.072), intercept 0.034 (SD 0.156) and ECE 0.031 (SD 0.010). For longer cadences such as half‐yearly to triennial, accuracy declined slightly to 0.840–0.844, sensitivity varied between 0.411 and 0.488, specificity from 0.930 to 0.951, precision from 0.652 to 0.691, NPV from 0.863 to 0.877, and F1 score from 0.508 to 0.550. Overall, calibration metrics trended towards degradation in extended cadences, particularly for the static model with the full set of secondary outcomes reported in Appendix S1.

### 3.3 Feature Importance

**FIGURE 2:**
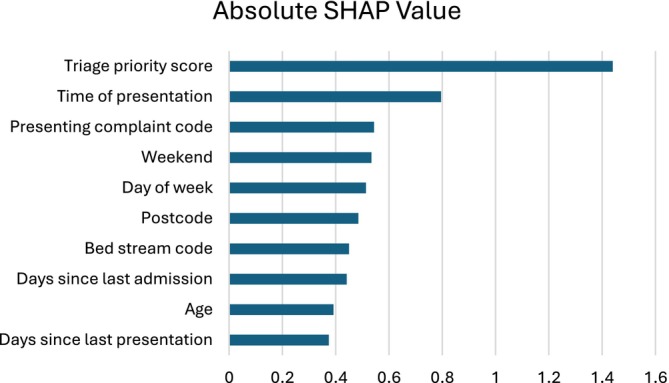
SHAP feature importance.

SHAP analysis, visualised in Figure 2, identified triage priority (1.439) as the primary predictor, followed by presentation timing (morning: 0.853; evening: 0.819; afternoon: 0.715) and presenting complaint codes (0.544). Temporal features (day: 0.434–0.513) and historical metrics (days since last presentation and admission: 0.374 and 0.441) contributed moderately.

## 4 Discussion

This simulation study evaluated an ensemble ML model for predicting inpatient admissions from triage data in a tertiary paediatric ED. Models that can accurately predict admissions from triage can forecast the upcoming bed demands across the hospital and improve resource allocation prior to patient medical review. The studied model utilises both structured features and NLP‐derived embeddings from triage notes to achieve a mean AUROC of 0.843 and an AMDBE of 2.57 over 5 years with weekly retraining. Retraining cadences of fortnightly and monthly also showed non‐inferior results with 50% and 75% less computational burden, respectively. This demonstrated robust discriminative performance with regular retraining over the 5‐year testing period, including the COVID‐19 pandemic.

These results align with prior research using ML for ED admission prediction. Existing models utilising triage data have reported AUROC values ranging from 0.81 to 0.93 [4, 7, 8]. In paediatric contexts, similar approaches have shown efficacy, with one investigation attaining an AUROC of 0.907 for related outcomes such as identifying emergency paediatric cases [15]. However, whilst AUROC assesses discrimination effectively, it may not adequately reflect operational utility; hence, the use of AMDBE as a clinically oriented metric. Although most publications prioritise discrimination metrics, AMDBE provides actionable insights for bed managers to guide resource allocation. AMDBE also demonstrated substantial variability even when AUROC appears stable. This highlights the importance of incorporating calibration‐focused metrics to ensure models support practical decision‐making in healthcare environments. The weekly model's average AMDBE of 2.57 compares favourably to aggregated error measures in comparable studies [7]. King et al. report a comparable metric, an eight‐hourly mean absolute error, achieving 4.0 over approximately 3 months in a simulated prediction pipeline for adult emergency admissions [7]. This model's improved calibration performance may be due to addressing seasonal and presentation variability challenges within the simulation or cohort‐related differences.

Marked covariate and concept drift emerged during the latter half of the study period, coinciding with the largest case numbers of COVID‐19 in Western Australia in early 2022. Whilst covariate distributions remained relatively stable until 2021, the domain‐classifier performance greatly improved from January 2022 onwards, achieving an AUROC of 0.92–0.99 when differentiating presentations from the 2022–2024 period from the initial training window of July 2018 to June 2019. Changes in the relationship between admission and the features of triage priority, presenting complaint, ED representations and ED bed stream had the largest impact. Importantly, this covariate drift translated into measurable degradation in the static model's calibration and, to a lesser extent, discrimination. Without retraining, AUROC drifted slightly (0.852 to 0.799); however, larger performance drift was observed in calibration intercepts and AMDBE, as observed admission rates exceeded the predicted admission demands. Such drift resulted in systematic underestimation of demand, which, without retraining, could have negative operational implications for bed management, surge preparedness and flow planning. In contrast, the comparative retraining simulations demonstrate that drift adaptation was effective. Weekly, fortnightly and monthly retraining mitigated both covariate and concept drift, maintaining stable AUROC and improving AMDBE, sustaining an acceptable calibration profile across the full test horizon. In contrast, longer cadences allowed drift to accumulate, resulting in miscalibration and predicted operational error. These findings highlight that in rapidly evolving clinical environments, frequent recalibration or retraining schedules are likely necessary to address covariate drift and sustain both model discrimination and calibration.

It is important to consider the computational cost of retraining this model when extrapolating the feasibility of different retraining cadences. Using four NVIDIA H100 GPUs and 64 CPU cores, a full retraining cycle would complete in approximately 5 min. This is substantially less demanding than the large‐scale training associated with frontier generative AI systems. This distinction is important, as resource burden remains a key barrier to ML and AI feasibility and adoption. Nonetheless, relative efficiency gains are meaningful, with monthly retraining requiring 25% of the annual compute of weekly retraining. Future research can consider performance‐based adaptive frameworks, in which retraining is initiated when a calibration metric such as AMDBE crosses a pre‐specified drift threshold. This would allow reduced retraining during stable periods whilst automatically escalating during rapid covariate shift, though monthly retraining may remain as a benchmark for a minimum retraining cadence frequency.

Even with frequent retraining to address covariate drift, higher presentation variability and DI exhibited strong correlations with weekly model error (*r* = 0.77 for standard deviation and *r* = 0.68 for ID), whereas admission rates showed negligible association. This indicates that fluctuations in patient volume, rather than admission prevalence, are a key contributor to diminished predictive accuracy during periods of volatility in this population. Given temporal features also had high importance in the SHAP analysis, temporal features show strong influence on model robustness in dynamic paediatric clinical settings [11, 12]. To mitigate this volatility‐driven error, future models could incorporate additional volume‐predictive features such as environmental or epidemiological indicators like school holiday periods.

Secondary metrics across different retraining cadences highlight the differential effects of concept drift on model performance. Classification thresholds reveal a conservative bias inherent to the model, with consistently low sensitivity and high specificity, reflecting its design to address class imbalance via Synthetic Minority Over‐sampling Technique [16]. Calibration metrics also show degradation with infrequent or no retraining, with the Brier score rising from 0.113 to 0.123 from weekly to static, expected calibration error (ECE) increases from 0.031 to 0.052, and the calibration intercept deviates further from the ideal of 0, moving from 0.034 to 0.321. The weekly retraining model's Brier score of 0.113 outperforms a comparable paediatric emergency model with Brier scores of 0.164–0.209 [15, 17]. Overall, these insights emphasise the need for regular model retraining and that whilst discrimination suffices for ranking, calibration is vital for aggregate forecasting and likely to be essential for clinical utility in an ED setting.

The clinical utility of this model should be considered within a staged translational context. The objective of this simulation study was to establish whether a well‐calibrated, temporally validated admission prediction model can be sustained across a multi‐year horizon inclusive of major disruptive events. Whether triage‐based admission prediction, available hours prior to clinician review, can meaningfully inform operational decisions surrounding bed allocation and staffing capacity remains to be established. Prospective clinical implementation is now required to determine whether this lead time translates into measurable operational benefit. Limitations.

The narrow study focus strengthened temporal analysis and understanding, though limitations include the single centre of data collection limiting external validity and lack of clinical implementation. The weekly data division (W1: days 1–7; W2: 8–15; W3: 16–22; W4: 23–end) introduces minor bias in W4 for months of varying length, such as January compared to February. Though this impact is consistent, limited, and enabled fair inter‐cadence performance comparisons. Finally, vital signs, whilst often reported in the literature, are not collected at triage in this centre, so are not included in this model.

## 5 Conclusion

This study explores concept drift in paediatric ED triage admission prediction and optimises performance via variable intervals of retraining across a 5‐year simulation. It describes and reports AMDBE, a calibration metric, which aligns with requirements of clinical application. AMDBE expressed more concept drift than traditionally reported measures such as AUROC, sensitivity and specificity. This highlights the importance of measuring and addressing both discrimination and calibration performance metrics. The model showed equivalent performance with weekly, fortnightly and monthly retraining, with monthly retraining requiring a quarter of the compute as compared to weekly retraining. Future research should focus on prospective clinical implementation to evaluate whether temporally validated admission prediction translates into measurable operational benefit, alongside external validation across a range of ED settings. Exploration of drift monitoring and performance triggered retraining could further improve or maintain performance whilst minimising computational burden, with this paper providing an initial benchmark for minimum retraining cadences.

## Funding

This work was supported by the Australian Government Research Training Programme and Noton Dale Medical Research Scholarship via the University of Notre Dame Australia, School of Medicine.

## Conflicts of Interest

The authors declare no conflicts of interest.

## Supporting information


**Figure S1:** Training structure.
**Table S1:** Secondary outcomes measures for each model.
**Table S2:** Ability to discriminate the testing group from the training group.
**Table S3:** Static model predicted admissions compared to actual admissions by decile per year.
**Table S4:** Five most impactful variables on performance drift by degree contributing to performance drift over time.
**Table S5:** Primary outcomes for five most frequently retraining models.
**Table S6:** Primary outcomes for the five least frequently retrained models.

**Appendix S2:** emm70271‐sup‐0002‐Appendicx_S2.docx.

## Data Availability

The data that support the findings of this study are available on request from the corresponding author. The data are not publicly available due to privacy or ethical restrictions.
