# Supplementary material for: Optimising Retraining Frequency for a Paediatric Emergency Department Admission Prediction Model: Development and Temporal Validation Using Real‐World Data
Source: Emerg Med Australas. 2026 May 6;38:e70271. doi: 10.1111/1742-6723.70271 (PMC13146150; doi:10.1111/1742-6723.70271)
Supplement: Supplementary file 1 — Figure S1: Training structure. Table S1: Secondary outcomes measures for each model. Table S2: Ability to discriminate the testing group from the training group. Table S3: Static model predicted admissions compared to actual admissions by decile per year. Table S4: Five most impactful variables on performance drift by degree contributing to performance drift over time. Table S5: Primary outcomes for five most frequently retraining models. Table S6: Primary outcomes for the five least frequently retrained models. [file EMM-38-0-s001.docx]

**Model Development and Training**

All simulations and data analysis was conducted on MERLIN, a secure high-performance cluster provided by the Stan Perron Charitable Foundation. All code was written by the research team in Python 3.9 within Spyder 6, with libraries including pandas, NumPy, SciPy, scikit-learn, XGBoost, PyTorch, TabNet, SHAP, matplotlib, and random seed 42 for reproducibility. An ensemble stacking framework was created, with base learners including mandatory XGBoost (XGB) and an optional multilayer perceptron (MLP), TabNet and logistic regression (LR), with a LR meta-learner to blend the ensemble. Hyperparameters were grid-searched on validation sets: XGBoost (number of estimators: 600-1,500; learning rate: 0.05-0.1; maximum tree depth: 7-10; column sample by tree: 0.8-0.9; subsample: 0.9 fixed; embeddings: yes/no); MLP (hidden layers: 128, 256; dropout rate: 0.2; learning rate: 0.001; maximum epochs: 30; early stopping patience: 6; batch size: 2,048); TabNet (n_d and n_a: 32; number of steps: 3; gamma: 1.3; maximum epochs: 100; early stopping patience: 12). Synthetic Minority Over-sampling Technique was used to address class imbalance for XGB, given a 4:1 class imbalance towards discharges. A post-hoc correction factor then adjusted probabilities based on training set calibration, recalculated each time the model was retrained.

NLP embeddings were generated from two triage free-text fields, triage description and nursing triage assessment, utilising a base BioClinicalBERT model. Initially, task-adaptive pre-training was performed via masked language modelling on the first year of data, with a batch size of 32, learning rate of 0.00005, and one epoch. Subsequently, simple contrastive sentence embedding fine-tuning was applied, constructing weakly supervised positive pairs via term frequency-inverse document frequency nearest-neighbour similarity. This used a minimum cosine similarity of 0.30, up to 20 neighbours per note, with fallback to identity pairs, a batch size of 64, a learning rate of 0.0002, and two epochs. Limiting the training window to the initial year of data ensured no NLP occurred with any data in the five-year testing period. Embeddings for all presentations were computed by encoding head and tail crops separately (maximum length 256 tokens), averaging the representations, and L2-normalising the resulting 768-dimensional vectors per field.

Data was divided temporally into four ‘weekly’ blocks for each month (W1: days 1-7; W2: 8-15; W3: 16-22; W4: 23-month end), yielding a total 48 weeks per year. Initial training and validation sets for all cadences and NLP included only data from July 1 2018 to June 30 2019, temporally separate to the testing set, to reduce bias. Simulations then used rolling windows from July 1, 2019, onward to June 30 2024. For training, a variable window was used with a minimum 44 weeks, maximum 92 weeks, depending on the amount of data available at the time of training to ensure the data was temporally separate to both the validation and testing set. The validation set was the rolling four-week period prior to the next test week at the time of retraining. Five-fold cross-validation generated out-of-fold predictions for meta-learner training on the validation set. For model and ensemble choice, lowest absolute mean daily bed error (AMDBE) on the validation set was used to select the best grid search and ensemble for the upcoming test period. Testing advanced by cadence-specific frequency, with retraining triggered by varying the retraining cadence parameter across separate runs. For example, fortnightly would retrain every two weeks, bimonthly would retrain every eight weeks and biennially would retrain every 96 weeks. These ‘weekly’ blocks created temporal separation between training, validation and test sets, while ensuring identical testing and training windows between all cadences for model performance comparison.

**Drift assessment methodology**

We evaluated both covariate drift across the static training window (Q3 2018–Q2 2019) compared to the five-year testing window (Q3 2019–Q2 2024). Covariate drift was assessed initially using an unsupervised principal component analysis (PCA) reconstruction error, quantifying reconstruction error from a PCA model trained exclusively on training-period covariates. A supervised domain classification was then performed by an XGBoost model trained to distinguish training encounters from each future quarter. Domain-classifier AUROC and associated feature importances were used to summarise the magnitude and principal drivers of distributional change.

Concept drift was assessed initially as described above by computing the primary and secondary model performance outcomes. Stratified decile calibration was then performed for the top five features with the most drift. Encounters were grouped within each period into deciles of predicted probability of admission and calculating the mean predicted risk, observed admission rate, and absolute difference. Bootstrap resampling was used to obtain uncertainty intervals for AUROC and recalibration parameters.

Training

Validation

Testing


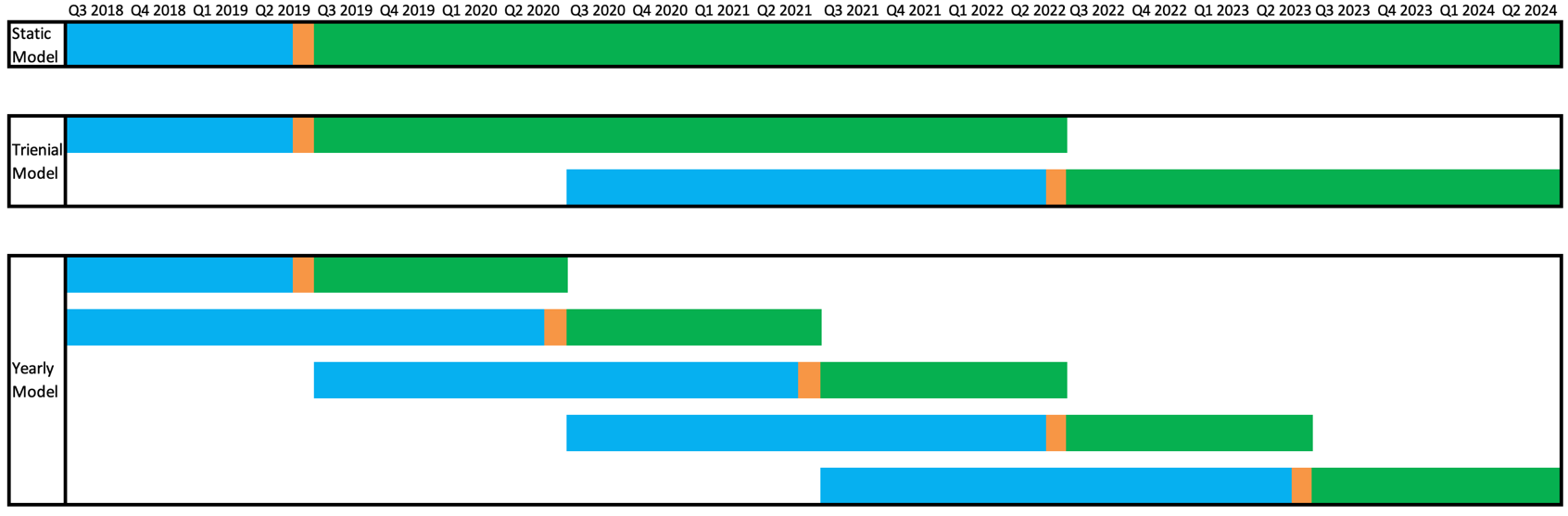


Figure S1: Training structure

| Retraining cadence | Accuracy | Sensitivity | Specificity | Precision | Negative predictive value | F1 score | Brier | Cal slope | Cal  intercept | Ece 10bin |
| --- | --- | --- | --- | --- | --- | --- | --- | --- | --- | --- |
| Weekly | 0.846 | 0.452 | 0.947 | 0.686 | 0.871 | 0.543 | 0.113 | 1.041 | 0.0342 | 0.0307 |
| Fortnightly | 0.846 | 0.452 | 0.947 | 0.686 | 0.871 | 0.543 | 0.113 | 1.043 | 0.0374 | 0.0308 |
| Monthly | 0.846 | 0.454 | 0.946 | 0.684 | 0.871 | 0.544 | 0.113 | 1.042 | 0.0343 | 0.0308 |
| Bimonthly | 0.845 | 0.453 | 0.945 | 0.681 | 0.871 | 0.542 | 0.114 | 1.040 | 0.0239 | 0.0321 |
| Trimonthly | 0.842 | 0.459 | 0.940 | 0.676 | 0.872 | 0.540 | 0.116 | 1.040 | 0.0193 | 0.0376 |
| Half-yearly | 0.844 | 0.443 | 0.946 | 0.685 | 0.869 | 0.532 | 0.115 | 1.033 | 0.0614 | 0.0365 |
| Yearly | 0.842 | 0.454 | 0.941 | 0.671 | 0.871 | 0.535 | 0.117 | 1.061 | 0.0256 | 0.0404 |
| Biennially | 0.841 | 0.411 | 0.951 | 0.691 | 0.863 | 0.508 | 0.119 | 1.063 | 0.175 | 0.0404 |
| Triennial | 0.840 | 0.488 | 0.930 | 0.652 | 0.877 | 0.550 | 0.117 | 1.028 | -0.0974 | 0.0465 |
| Static | 0.836 | 0.355 | 0.960 | 0.714 | 0.853 | 0.458 | 0.123 | 1.002 | 0.321 | 0.0519 |

Table S1: Secondary outcomes measures for each model

**Covariate drift results**

Unsupervised PCA reconstruction error suggested modest covariate drift between 2019 and 2021. Mean reconstruction errors remained low with increased variability during 2022–2023, consistent with emerging changes in the joint covariate distribution. In contrast, supervised domain-classification more clearly demonstrated temporal divergence as the XGBoost AUROC increased from moderate separability in 2019–2020 to values above 0.98 from 2022 onward as seen in Table S2. This indicates that encounters in later years were almost perfectly distinguishable from the original training set. Feature-importance profiles highlighted large shifts in representation features (‘days since last presentation’ and ‘presentations in the last 12 months’), ED bed allocation, trigae priority and presenting-complaint codes relative to admissions. This can be seen in Table S3 and Table S4.

Stratified decile calibration corroborated these patterns: for drift-sensitive features such as days since last presentation, differences between observed and predicted admission rates in mid- and high-risk deciles widened steadily from 2022 to 2024. While relative risk ordering remained largely preserved in the static model (calibration slopes remained close to 1), the absolute mapping between predictors and admission shifted. These results emphasise that even apparently stable AUROC can mask clinically important drift in calibration and aggregate bed-demand accuracy.

| **Year** | **Domain AUROC** | **Interpretation** |
| --- | --- | --- |
| 2019 | 0.63 | Mild drift |
| 2020 | 0.7 | Moderate drift |
| 2021 | 0.76 | Moderate drift |
| 2022 | 0.98 | Extreme drift |
| 2023 | 0.99 | Extreme drift |
| 2024 | 0.99 | Extreme drift |

Table S2: Ability to discriminate the testing group from the training group

| **Year** | **Decile** | **Mean Predicted Rate of admission** | **Observed Admission Rate** | **Difference** |
| --- | --- | --- | --- | --- |
| **2019** | 1 | 0.031 | 0.013 | 0.018 |
|  | 5 | 0.196 | 0.162 | 0.034 |
|  | 10 | 0.682 | 0.752 | -0.070 |
| **2020** | 1 | 0.032 | 0.019 | 0.013 |
|  | 5 | 0.192 | 0.199 | -0.007 |
|  | 10 | 0.664 | 0.729 | -0.065 |
| **2021** | 1 | 0.033 | 0.02 | 0.013 |
|  | 5 | 0.194 | 0.199 | -0.005 |
|  | 10 | 0.661 | 0.72 | -0.059 |
| **2022** | 1 | 0.029 | 0.036 | -0.007 |
|  | 5 | 0.177 | 0.255 | -0.078 |
|  | 10 | 0.568 | 0.689 | -0.121 |
| **2023** | 1 | 0.03 | 0.039 | -0.009 |
|  | 5 | 0.177 | 0.258 | -0.081 |
|  | 10 | 0.583 | 0.705 | -0.122 |
| **2024** | 1 | 0.03 | 0.034 | -0.004 |
|  | 5 | 0.176 | 0.233 | -0.057 |
|  | 10 | 0.578 | 0.701 | -0.123 |

Table S3: Static model predicted admissions compared to actual admissions by decile per year

| **Feature** | **Year** | **Decile** | **Mean Predicted Rate of admission** | **Observed Admission Rate** | **Difference** | **Mean Difference** |
| --- | --- | --- | --- | --- | --- | --- |
| Days since last presentation | 2019 | 1 | 0.028 | 0.005 | 0.024 | -0.034 |
|  |  | 5 | 0.090 | 0.163 | -0.074 |  |
|  |  | 10 | 0.633 | 0.684 | -0.051 |  |
|  | 2020 | 1 | 0.031 | 0.024 | 0.008 | -0.019 |
|  |  | 5 | 0.123 | 0.232 | -0.109 |  |
|  |  | 10 | 0.646 | 0.600 | 0.046 |  |
|  | 2021 | 1 | 0.033 | 0.035 | -0.002 | 0.018 |
|  |  | 5 | 0.168 | 0.156 | 0.011 |  |
|  |  | 10 | 0.674 | 0.629 | 0.045 |  |
|  | 2022 | 1 | 0.030 | 0.068 | -0.038 | -0.091 |
|  |  | 5 | 0.071 | 0.229 | -0.158 |  |
|  |  | 10 | 0.552 | 0.628 | -0.076 |  |
|  | 2023 | 1 | 0.031 | 0.060 | -0.029 | -0.109 |
|  |  | 5 | 0.081 | 0.321 | -0.240 |  |
|  |  | 10 | 0.587 | 0.645 | -0.058 |  |
|  | 2024 | 1 | 0.031 | 0.057 | -0.026 | -0.096 |
|  |  | 5 | 0.082 | 0.267 | -0.185 |  |
|  |  | 10 | 0.558 | 0.636 | -0.077 |  |
| ED bed stream | 2019 | 1 | 0.037 | 0.030 | 0.007 | -0.026 |
|  |  | 5 | 0.124 | 0.132 | -0.008 |  |
|  |  | 10 | 0.506 | 0.583 | -0.076 |  |
|  | 2020 | 1 | 0.039 | 0.033 | 0.006 | -0.022 |
|  |  | 5 | 0.123 | 0.134 | -0.011 |  |
|  |  | 10 | 0.504 | 0.566 | -0.062 |  |
|  | 2021 | 1 | 0.041 | 0.036 | 0.004 | -0.004 |
|  |  | 5 | 0.155 | 0.141 | 0.014 |  |
|  |  | 10 | 0.484 | 0.515 | -0.031 |  |
|  | 2022 | 1 | 0.030 | 0.039 | -0.009 | -0.069 |
|  |  | 5 | 0.054 | 0.118 | -0.064 |  |
|  |  | 10 | 0.538 | 0.672 | -0.135 |  |
|  | 2023 | 1 | 0.030 | 0.038 | -0.008 | -0.059 |
|  |  | 5 | 0.058 | 0.141 | -0.083 |  |
|  |  | 10 | 0.557 | 0.644 | -0.087 |  |
|  | 2024 | 1 | 0.030 | 0.034 | -0.003 | -0.052 |
|  |  | 5 | 0.062 | 0.143 | -0.082 |  |
|  |  | 10 | 0.558 | 0.630 | -0.072 |  |
| Triage priority score | 2019 | 1 | 0.055 | 0.061 | -0.006 | -0.016 |
|  |  | 5 | 0.163 | 0.160 | 0.003 |  |
|  |  | 10 | 0.439 | 0.485 | -0.046 |  |
|  | 2020 | 1 | 0.054 | 0.059 | -0.005 | -0.013 |
|  |  | 5 | 0.161 | 0.140 | 0.021 |  |
|  |  | 10 | 0.435 | 0.489 | -0.054 |  |
|  | 2021 | 1 | 0.060 | 0.061 | -0.001 | 0.001 |
|  |  | 5 | 0.179 | 0.147 | 0.032 |  |
|  |  | 10 | 0.433 | 0.462 | -0.029 |  |
|  | 2022 | 1 | 0.036 | 0.099 | -0.063 | -0.078 |
|  |  | 5 | 0.096 | 0.177 | -0.081 |  |
|  |  | 10 | 0.326 | 0.415 | -0.089 |  |
|  | 2023 | 1 | 0.037 | 0.112 | -0.075 | -0.067 |
|  |  | 5 | 0.106 | 0.183 | -0.077 |  |
|  |  | 10 | 0.355 | 0.406 | -0.051 |  |
|  | 2024 | 1 | 0.037 | 0.104 | -0.066 | -0.063 |
|  |  | 5 | 0.107 | 0.175 | -0.069 |  |
|  |  | 10 | 0.368 | 0.421 | -0.053 |  |
| Presentations in the last12 months | 2019 | 1 | 0.031 | 0.017 | 0.014 | -0.022 |
|  |  | 5 | 0.068 | 0.080 | -0.012 |  |
|  |  | 10 | 0.673 | 0.741 | -0.068 |  |
|  | 2020 | 1 | 0.032 | 0.018 | 0.014 | -0.014 |
|  |  | 5 | 0.071 | 0.086 | -0.015 |  |
|  |  | 10 | 0.662 | 0.703 | -0.041 |  |
|  | 2021 | 1 | 0.032 | 0.017 | 0.015 | -0.007 |
|  |  | 5 | 0.073 | 0.087 | -0.014 |  |
|  |  | 10 | 0.670 | 0.693 | -0.023 |  |
|  | 2022 | 1 | 0.030 | 0.036 | -0.007 | -0.065 |
|  |  | 5 | 0.047 | 0.106 | -0.060 |  |
|  |  | 10 | 0.550 | 0.679 | -0.129 |  |
|  | 2023 | 1 | 0.030 | 0.040 | -0.010 | -0.063 |
|  |  | 5 | 0.054 | 0.117 | -0.063 |  |
|  |  | 10 | 0.568 | 0.685 | -0.117 |  |
|  | 2024 | 1 | 0.030 | 0.038 | -0.008 | -0.062 |
|  |  | 5 | 0.057 | 0.116 | -0.059 |  |
|  |  | 10 | 0.565 | 0.684 | -0.119 |  |
| Presenting complaint code | 2019 | 1 | 0.031 | 0.014 | 0.017 | -0.010 |
|  |  | 5 | 0.067 | 0.079 | -0.012 |  |
|  |  | 10 | 0.646 | 0.682 | -0.036 |  |
|  | 2020 | 1 | 0.032 | 0.022 | 0.010 | -0.005 |
|  |  | 5 | 0.072 | 0.085 | -0.014 |  |
|  |  | 10 | 0.642 | 0.653 | -0.011 |  |
|  | 2021 | 1 | 0.032 | 0.022 | 0.010 | 0.000 |
|  |  | 5 | 0.077 | 0.091 | -0.014 |  |
|  |  | 10 | 0.662 | 0.658 | 0.004 |  |
|  | 2022 | 1 | 0.030 | 0.043 | -0.014 | -0.064 |
|  |  | 5 | 0.049 | 0.114 | -0.065 |  |
|  |  | 10 | 0.531 | 0.645 | -0.114 |  |
|  | 2023 | 1 | 0.031 | 0.050 | -0.019 | -0.062 |
|  |  | 5 | 0.059 | 0.122 | -0.063 |  |
|  |  | 10 | 0.542 | 0.646 | -0.103 |  |
|  | 2024 | 1 | 0.031 | 0.046 | -0.016 | -0.059 |
|  |  | 5 | 0.060 | 0.125 | -0.065 |  |
|  |  | 10 | 0.534 | 0.632 | -0.097 |  |

Table S4: Five most impactful variables on performance drift by degree contributing to performance drift over time

Full primary outcomes for each model across every week of testing can be found below.

|  | **Weekly** | | **Fortnightly** | | **Monthly** | | **Bimonthly** | | **Trimonthly** | |
| --- | --- | --- | --- | --- | --- | --- | --- | --- | --- | --- |
| **Date** | **AMDBE** | **AUROC** | **AMDBE** | **AUROC** | **AMDBE** | **AUROC** | **AMDBE** | **AUROC** | **AMDBE** | **AUROC** |
| 2019-07-W1 | 2.714 | 0.852 | 2.655 | 0.857 | 2.668 | 0.856 | 3.483 | 0.852 | 2.688 | 0.856 |
| 2019-07-W2 | 3.453 | 0.857 | 3.801 | 0.866 | 3.765 | 0.866 | 3.811 | 0.863 | 3.776 | 0.866 |
| 2019-07-W3 | 1.350 | 0.867 | 1.705 | 0.874 | 1.715 | 0.871 | 1.138 | 0.863 | 1.707 | 0.870 |
| 2019-07-W4 | 1.438 | 0.850 | 1.502 | 0.849 | 1.272 | 0.851 | 1.138 | 0.853 | 1.204 | 0.851 |
| 2019-08-W1 | 2.769 | 0.858 | 2.748 | 0.858 | 2.843 | 0.859 | 3.262 | 0.855 | 3.007 | 0.857 |
| 2019-08-W2 | 2.372 | 0.843 | 4.121 | 0.854 | 4.071 | 0.855 | 3.100 | 0.856 | 3.884 | 0.854 |
| 2019-08-W3 | 3.399 | 0.862 | 3.399 | 0.862 | 2.652 | 0.874 | 3.293 | 0.868 | 2.752 | 0.873 |
| 2019-08-W4 | 2.769 | 0.855 | 3.037 | 0.852 | 2.142 | 0.861 | 2.701 | 0.856 | 2.222 | 0.857 |
| 2019-09-W1 | 5.156 | 0.847 | 5.156 | 0.847 | 5.156 | 0.847 | 5.156 | 0.847 | 4.402 | 0.860 |
| 2019-09-W2 | 1.246 | 0.846 | 1.421 | 0.846 | 1.421 | 0.846 | 1.421 | 0.846 | 1.318 | 0.851 |
| 2019-09-W3 | 4.550 | 0.864 | 4.536 | 0.865 | 5.214 | 0.855 | 5.214 | 0.855 | 4.362 | 0.866 |
| 2019-09-W4 | 2.457 | 0.846 | 2.172 | 0.850 | 2.563 | 0.836 | 2.563 | 0.836 | 2.524 | 0.853 |
| 2019-10-W1 | 1.723 | 0.854 | 1.702 | 0.852 | 1.696 | 0.853 | 1.202 | 0.841 | 1.754 | 0.854 |
| 2019-10-W2 | 4.241 | 0.831 | 4.480 | 0.831 | 4.388 | 0.832 | 4.889 | 0.827 | 4.385 | 0.831 |
| 2019-10-W3 | 3.004 | 0.872 | 2.961 | 0.873 | 3.029 | 0.873 | 3.459 | 0.867 | 3.052 | 0.873 |
| 2019-10-W4 | 2.128 | 0.866 | 2.113 | 0.865 | 2.089 | 0.865 | 1.962 | 0.863 | 2.107 | 0.865 |
| 2019-11-W1 | 4.118 | 0.865 | 4.196 | 0.865 | 4.210 | 0.864 | 4.132 | 0.865 | 3.957 | 0.861 |
| 2019-11-W2 | 1.126 | 0.860 | 1.316 | 0.865 | 1.107 | 0.860 | 1.315 | 0.866 | 1.359 | 0.864 |
| 2019-11-W3 | 1.412 | 0.865 | 1.412 | 0.865 | 1.294 | 0.866 | 1.238 | 0.869 | 1.403 | 0.865 |
| 2019-11-W4 | 1.290 | 0.852 | 1.209 | 0.853 | 1.331 | 0.851 | 1.437 | 0.851 | 1.597 | 0.852 |
| 2019-12-W1 | 0.794 | 0.861 | 0.794 | 0.861 | 0.794 | 0.861 | 1.075 | 0.870 | 0.959 | 0.866 |
| 2019-12-W2 | 1.591 | 0.817 | 1.171 | 0.824 | 1.171 | 0.824 | 1.246 | 0.838 | 1.292 | 0.832 |
| 2019-12-W3 | 1.302 | 0.835 | 1.302 | 0.835 | 1.100 | 0.837 | 0.946 | 0.845 | 0.955 | 0.841 |
| 2019-12-W4 | 2.799 | 0.806 | 2.341 | 0.810 | 2.823 | 0.808 | 2.880 | 0.813 | 2.764 | 0.813 |
| 2020-01-W1 | 2.095 | 0.842 | 2.095 | 0.842 | 2.095 | 0.842 | 2.095 | 0.842 | 2.095 | 0.842 |
| 2020-01-W2 | 1.041 | 0.840 | 0.894 | 0.839 | 0.894 | 0.839 | 0.894 | 0.839 | 0.894 | 0.839 |
| 2020-01-W3 | 4.763 | 0.831 | 4.763 | 0.831 | 4.285 | 0.834 | 4.285 | 0.834 | 4.285 | 0.834 |
| 2020-01-W4 | 1.541 | 0.843 | 1.613 | 0.844 | 1.186 | 0.842 | 1.186 | 0.842 | 1.186 | 0.842 |
| 2020-02-W1 | 2.351 | 0.865 | 2.319 | 0.866 | 2.391 | 0.866 | 2.080 | 0.860 | 2.080 | 0.860 |
| 2020-02-W2 | 1.673 | 0.866 | 1.209 | 0.867 | 1.234 | 0.867 | 1.472 | 0.859 | 1.472 | 0.859 |
| 2020-02-W3 | 3.897 | 0.868 | 3.829 | 0.866 | 3.666 | 0.867 | 3.774 | 0.862 | 3.774 | 0.862 |
| 2020-02-W4 | 2.375 | 0.853 | 2.453 | 0.848 | 2.445 | 0.853 | 2.561 | 0.848 | 2.561 | 0.848 |
| 2020-03-W1 | 2.519 | 0.857 | 2.528 | 0.858 | 2.502 | 0.858 | 2.471 | 0.858 | 2.292 | 0.846 |
| 2020-03-W2 | 2.069 | 0.856 | 2.589 | 0.862 | 2.459 | 0.863 | 2.508 | 0.862 | 1.380 | 0.852 |
| 2020-03-W3 | 2.675 | 0.841 | 2.571 | 0.841 | 2.616 | 0.842 | 2.584 | 0.841 | 3.360 | 0.829 |
| 2020-03-W4 | 0.809 | 0.856 | 0.793 | 0.854 | 0.731 | 0.856 | 0.693 | 0.857 | 0.657 | 0.840 |
| 2020-04-W1 | 1.048 | 0.833 | 1.075 | 0.833 | 1.030 | 0.832 | 0.960 | 0.830 | 1.065 | 0.833 |
| 2020-04-W2 | 2.502 | 0.838 | 2.603 | 0.841 | 2.617 | 0.840 | 2.589 | 0.841 | 2.543 | 0.841 |
| 2020-04-W3 | 1.780 | 0.838 | 1.783 | 0.836 | 1.830 | 0.832 | 1.589 | 0.830 | 1.861 | 0.832 |
| 2020-04-W4 | 3.090 | 0.825 | 2.672 | 0.839 | 2.727 | 0.841 | 2.717 | 0.830 | 2.728 | 0.841 |
| 2020-05-W1 | 2.152 | 0.832 | 2.181 | 0.833 | 2.134 | 0.832 | 2.132 | 0.834 | 2.439 | 0.831 |
| 2020-05-W2 | 2.604 | 0.834 | 2.501 | 0.832 | 2.600 | 0.832 | 2.529 | 0.833 | 2.804 | 0.834 |
| 2020-05-W3 | 0.949 | 0.852 | 0.827 | 0.850 | 1.000 | 0.853 | 0.958 | 0.853 | 1.282 | 0.851 |
| 2020-05-W4 | 1.430 | 0.857 | 1.423 | 0.853 | 1.467 | 0.856 | 1.425 | 0.856 | 1.726 | 0.858 |
| 2020-06-W1 | 2.134 | 0.847 | 2.153 | 0.845 | 2.076 | 0.846 | 1.940 | 0.848 | 1.589 | 0.845 |
| 2020-06-W2 | 1.585 | 0.857 | 2.099 | 0.861 | 2.152 | 0.861 | 1.853 | 0.864 | 1.454 | 0.859 |
| 2020-06-W3 | 0.782 | 0.851 | 0.813 | 0.845 | 0.908 | 0.853 | 0.724 | 0.854 | 0.734 | 0.854 |
| 2020-06-W4 | 4.342 | 0.839 | 4.329 | 0.833 | 3.643 | 0.840 | 3.652 | 0.841 | 4.235 | 0.837 |
| 2020-07-W1 | 1.058 | 0.846 | 0.994 | 0.849 | 0.981 | 0.850 | 0.994 | 0.849 | 0.914 | 0.849 |
| 2020-07-W2 | 3.570 | 0.845 | 3.664 | 0.846 | 3.574 | 0.850 | 3.695 | 0.846 | 3.626 | 0.845 |
| 2020-07-W3 | 1.345 | 0.853 | 1.254 | 0.852 | 1.318 | 0.853 | 1.141 | 0.851 | 1.127 | 0.852 |
| 2020-07-W4 | 1.002 | 0.841 | 1.113 | 0.842 | 1.040 | 0.841 | 0.982 | 0.843 | 1.206 | 0.842 |
| 2020-08-W1 | 1.663 | 0.854 | 1.663 | 0.854 | 1.663 | 0.854 | 1.756 | 0.849 | 1.676 | 0.848 |
| 2020-08-W2 | 2.938 | 0.822 | 2.680 | 0.827 | 2.680 | 0.827 | 2.331 | 0.840 | 2.337 | 0.840 |
| 2020-08-W3 | 3.101 | 0.852 | 3.101 | 0.852 | 2.820 | 0.858 | 2.599 | 0.861 | 2.591 | 0.860 |
| 2020-08-W4 | 12.742 | 0.826 | 12.753 | 0.835 | 12.945 | 0.835 | 12.843 | 0.842 | 12.759 | 0.842 |
| 2020-09-W1 | 2.053 | 0.842 | 2.053 | 0.842 | 1.925 | 0.852 | 2.053 | 0.842 | 1.927 | 0.853 |
| 2020-09-W2 | 3.137 | 0.826 | 2.499 | 0.839 | 2.724 | 0.850 | 2.499 | 0.839 | 2.326 | 0.847 |
| 2020-09-W3 | 2.504 | 0.852 | 2.504 | 0.852 | 2.231 | 0.871 | 2.590 | 0.852 | 2.350 | 0.871 |
| 2020-09-W4 | 1.462 | 0.827 | 1.547 | 0.828 | 1.299 | 0.855 | 1.533 | 0.846 | 1.405 | 0.850 |
| 2020-10-W1 | 4.091 | 0.823 | 4.067 | 0.823 | 4.132 | 0.823 | 4.270 | 0.799 | 3.790 | 0.819 |
| 2020-10-W2 | 2.364 | 0.831 | 2.431 | 0.841 | 2.466 | 0.841 | 2.787 | 0.820 | 2.658 | 0.832 |
| 2020-10-W3 | 3.493 | 0.861 | 3.477 | 0.860 | 3.470 | 0.861 | 4.136 | 0.840 | 3.760 | 0.858 |
| 2020-10-W4 | 3.035 | 0.829 | 2.995 | 0.830 | 3.001 | 0.830 | 3.554 | 0.815 | 3.085 | 0.827 |
| 2020-11-W1 | 3.112 | 0.848 | 3.165 | 0.847 | 3.210 | 0.848 | 3.118 | 0.847 | 3.663 | 0.851 |
| 2020-11-W2 | 0.746 | 0.831 | 1.094 | 0.829 | 1.126 | 0.829 | 1.172 | 0.829 | 1.143 | 0.836 |
| 2020-11-W3 | 6.410 | 0.854 | 6.646 | 0.854 | 6.347 | 0.850 | 6.228 | 0.849 | 7.279 | 0.857 |
| 2020-11-W4 | 2.704 | 0.827 | 2.894 | 0.826 | 2.493 | 0.829 | 2.573 | 0.826 | 3.224 | 0.821 |
| 2020-12-W1 | 3.914 | 0.844 | 3.662 | 0.843 | 3.779 | 0.843 | 3.985 | 0.846 | 4.534 | 0.838 |
| 2020-12-W2 | 3.947 | 0.843 | 3.715 | 0.843 | 3.907 | 0.843 | 4.288 | 0.843 | 4.378 | 0.851 |
| 2020-12-W3 | 2.572 | 0.826 | 3.417 | 0.833 | 3.413 | 0.832 | 3.087 | 0.831 | 4.251 | 0.830 |
| 2020-12-W4 | 4.839 | 0.853 | 5.048 | 0.851 | 4.985 | 0.853 | 4.949 | 0.850 | 5.497 | 0.850 |
| 2021-01-W1 | 2.936 | 0.850 | 2.936 | 0.850 | 2.936 | 0.850 | 2.936 | 0.850 | 2.936 | 0.850 |
| 2021-01-W2 | 1.649 | 0.835 | 1.417 | 0.837 | 1.417 | 0.837 | 1.417 | 0.837 | 1.417 | 0.837 |
| 2021-01-W3 | 4.356 | 0.863 | 4.259 | 0.851 | 4.319 | 0.849 | 4.319 | 0.849 | 4.319 | 0.849 |
| 2021-01-W4 | 3.975 | 0.838 | 3.760 | 0.825 | 3.927 | 0.826 | 3.927 | 0.826 | 3.927 | 0.826 |
| 2021-02-W1 | 1.528 | 0.844 | 1.528 | 0.844 | 1.528 | 0.844 | 0.947 | 0.842 | 0.947 | 0.842 |
| 2021-02-W2 | 2.176 | 0.850 | 1.964 | 0.847 | 1.964 | 0.847 | 1.584 | 0.835 | 1.584 | 0.835 |
| 2021-02-W3 | 2.503 | 0.864 | 2.583 | 0.868 | 2.797 | 0.867 | 2.395 | 0.859 | 2.395 | 0.859 |
| 2021-02-W4 | 1.814 | 0.835 | 1.658 | 0.834 | 2.044 | 0.833 | 2.143 | 0.826 | 2.143 | 0.826 |
| 2021-03-W1 | 2.123 | 0.863 | 2.123 | 0.863 | 2.123 | 0.863 | 2.123 | 0.863 | 2.192 | 0.874 |
| 2021-03-W2 | 3.030 | 0.844 | 4.371 | 0.844 | 4.371 | 0.844 | 4.371 | 0.844 | 3.415 | 0.848 |
| 2021-03-W3 | 1.621 | 0.852 | 1.555 | 0.851 | 1.857 | 0.832 | 1.857 | 0.832 | 2.232 | 0.837 |
| 2021-03-W4 | 6.967 | 0.862 | 6.706 | 0.860 | 7.599 | 0.839 | 7.599 | 0.839 | 6.580 | 0.853 |
| 2021-04-W1 | 2.505 | 0.829 | 2.505 | 0.829 | 2.505 | 0.829 | 1.804 | 0.817 | 2.505 | 0.829 |
| 2021-04-W2 | 1.260 | 0.857 | 1.058 | 0.849 | 1.058 | 0.849 | 1.191 | 0.838 | 1.058 | 0.849 |
| 2021-04-W3 | 1.906 | 0.861 | 1.712 | 0.863 | 1.513 | 0.852 | 2.363 | 0.859 | 1.513 | 0.852 |
| 2021-04-W4 | 2.588 | 0.834 | 2.226 | 0.831 | 1.760 | 0.826 | 2.594 | 0.814 | 1.760 | 0.826 |
| 2021-05-W1 | 1.215 | 0.842 | 1.215 | 0.842 | 1.215 | 0.842 | 1.215 | 0.842 | 1.131 | 0.841 |
| 2021-05-W2 | 0.819 | 0.882 | 1.645 | 0.867 | 1.645 | 0.867 | 1.645 | 0.867 | 1.547 | 0.861 |
| 2021-05-W3 | 2.980 | 0.852 | 3.272 | 0.852 | 2.457 | 0.843 | 2.457 | 0.843 | 2.673 | 0.837 |
| 2021-05-W4 | 1.027 | 0.829 | 2.156 | 0.844 | 1.447 | 0.826 | 1.447 | 0.826 | 1.210 | 0.824 |
| 2021-06-W1 | 2.185 | 0.845 | 2.185 | 0.845 | 2.185 | 0.845 | 1.915 | 0.847 | 2.118 | 0.836 |
| 2021-06-W2 | 1.813 | 0.847 | 1.270 | 0.827 | 1.270 | 0.827 | 1.718 | 0.836 | 1.660 | 0.830 |
| 2021-06-W3 | 4.519 | 0.871 | 4.519 | 0.871 | 4.060 | 0.862 | 4.961 | 0.872 | 4.844 | 0.868 |
| 2021-06-W4 | 1.690 | 0.849 | 1.661 | 0.841 | 1.936 | 0.835 | 1.866 | 0.839 | 1.779 | 0.837 |
| 2021-07-W1 | 0.868 | 0.839 | 0.868 | 0.839 | 0.868 | 0.839 | 0.868 | 0.839 | 0.868 | 0.839 |
| 2021-07-W2 | 1.278 | 0.844 | 0.701 | 0.830 | 0.701 | 0.830 | 0.701 | 0.830 | 0.701 | 0.830 |
| 2021-07-W3 | 1.602 | 0.823 | 1.602 | 0.823 | 1.672 | 0.821 | 1.672 | 0.821 | 1.672 | 0.821 |
| 2021-07-W4 | 9.660 | 0.857 | 7.495 | 0.855 | 7.887 | 0.852 | 7.887 | 0.852 | 7.887 | 0.852 |
| 2021-08-W1 | 9.491 | 0.854 | 9.491 | 0.854 | 9.491 | 0.854 | 10.391 | 0.850 | 10.391 | 0.850 |
| 2021-08-W2 | 12.258 | 0.855 | 11.602 | 0.842 | 11.602 | 0.842 | 11.795 | 0.839 | 11.795 | 0.839 |
| 2021-08-W3 | 7.408 | 0.856 | 7.408 | 0.856 | 7.832 | 0.857 | 8.037 | 0.855 | 8.037 | 0.855 |
| 2021-08-W4 | 7.593 | 0.854 | 7.549 | 0.856 | 8.070 | 0.853 | 8.479 | 0.850 | 8.479 | 0.850 |
| 2021-09-W1 | 1.701 | 0.837 | 1.701 | 0.837 | 1.701 | 0.837 | 1.701 | 0.837 | 1.613 | 0.833 |
| 2021-09-W2 | 2.103 | 0.855 | 2.073 | 0.858 | 2.073 | 0.858 | 2.073 | 0.858 | 2.721 | 0.856 |
| 2021-09-W3 | 4.443 | 0.855 | 4.443 | 0.855 | 4.619 | 0.848 | 4.619 | 0.848 | 5.313 | 0.855 |
| 2021-09-W4 | 1.751 | 0.834 | 1.731 | 0.836 | 1.372 | 0.836 | 1.372 | 0.836 | 1.994 | 0.842 |
| 2021-10-W1 | 3.276 | 0.838 | 3.276 | 0.838 | 3.276 | 0.838 | 2.520 | 0.826 | 3.276 | 0.838 |
| 2021-10-W2 | 1.688 | 0.874 | 1.944 | 0.876 | 1.944 | 0.876 | 1.900 | 0.852 | 1.944 | 0.876 |
| 2021-10-W3 | 1.453 | 0.810 | 1.453 | 0.810 | 1.894 | 0.830 | 1.236 | 0.812 | 1.894 | 0.830 |
| 2021-10-W4 | 2.169 | 0.859 | 1.462 | 0.848 | 2.305 | 0.853 | 1.729 | 0.846 | 2.305 | 0.853 |
| 2021-11-W1 | 5.502 | 0.844 | 6.363 | 0.823 | 5.525 | 0.844 | 6.363 | 0.823 | 5.316 | 0.841 |
| 2021-11-W2 | 5.709 | 0.830 | 5.396 | 0.818 | 5.402 | 0.821 | 5.396 | 0.818 | 5.616 | 0.823 |
| 2021-11-W3 | 2.866 | 0.849 | 2.866 | 0.849 | 3.823 | 0.864 | 3.872 | 0.855 | 4.309 | 0.867 |
| 2021-11-W4 | 6.024 | 0.830 | 4.324 | 0.805 | 5.778 | 0.826 | 5.531 | 0.816 | 6.465 | 0.830 |
| 2021-12-W1 | 1.475 | 0.842 | 1.475 | 0.842 | 1.475 | 0.842 | 3.133 | 0.838 | 3.267 | 0.838 |
| 2021-12-W2 | 3.120 | 0.846 | 3.197 | 0.847 | 3.197 | 0.847 | 4.808 | 0.847 | 5.694 | 0.852 |
| 2021-12-W3 | 2.605 | 0.820 | 2.605 | 0.820 | 2.751 | 0.822 | 1.515 | 0.813 | 1.686 | 0.830 |
| 2021-12-W4 | 1.116 | 0.834 | 1.210 | 0.838 | 1.140 | 0.833 | 2.067 | 0.837 | 2.465 | 0.849 |
| 2022-01-W1 | 4.412 | 0.823 | 4.412 | 0.823 | 4.412 | 0.823 | 4.412 | 0.823 | 4.412 | 0.823 |
| 2022-01-W2 | 0.877 | 0.821 | 1.939 | 0.818 | 1.939 | 0.818 | 1.939 | 0.818 | 1.939 | 0.818 |
| 2022-01-W3 | 1.980 | 0.816 | 1.980 | 0.816 | 1.875 | 0.825 | 1.875 | 0.825 | 1.875 | 0.825 |
| 2022-01-W4 | 1.635 | 0.816 | 2.089 | 0.827 | 1.663 | 0.822 | 1.663 | 0.822 | 1.663 | 0.822 |
| 2022-02-W1 | 2.528 | 0.858 | 2.216 | 0.859 | 2.635 | 0.858 | 4.523 | 0.836 | 4.523 | 0.836 |
| 2022-02-W2 | 1.429 | 0.783 | 8.016 | 0.862 | 8.733 | 0.861 | 11.711 | 0.831 | 11.711 | 0.831 |
| 2022-02-W3 | 1.908 | 0.803 | 2.283 | 0.802 | 7.209 | 0.794 | 10.118 | 0.779 | 10.118 | 0.779 |
| 2022-02-W4 | 4.678 | 0.856 | 4.680 | 0.855 | 4.431 | 0.837 | 6.707 | 0.821 | 6.707 | 0.821 |
| 2022-03-W1 | 0.972 | 0.819 | 1.039 | 0.835 | 0.782 | 0.834 | 1.103 | 0.834 | 8.828 | 0.797 |
| 2022-03-W2 | 1.829 | 0.796 | 3.011 | 0.812 | 3.383 | 0.812 | 2.960 | 0.813 | 7.811 | 0.763 |
| 2022-03-W3 | 2.500 | 0.820 | 2.171 | 0.834 | 2.934 | 0.831 | 2.564 | 0.831 | 9.487 | 0.780 |
| 2022-03-W4 | 1.104 | 0.845 | 1.108 | 0.846 | 3.033 | 0.843 | 2.614 | 0.844 | 6.369 | 0.810 |
| 2022-04-W1 | 2.991 | 0.811 | 3.048 | 0.812 | 2.748 | 0.811 | 1.453 | 0.802 | 2.966 | 0.812 |
| 2022-04-W2 | 1.938 | 0.839 | 2.280 | 0.835 | 2.121 | 0.831 | 1.505 | 0.838 | 2.325 | 0.831 |
| 2022-04-W3 | 4.254 | 0.833 | 4.254 | 0.833 | 4.536 | 0.842 | 2.306 | 0.851 | 4.747 | 0.843 |
| 2022-04-W4 | 2.814 | 0.817 | 3.037 | 0.824 | 3.058 | 0.844 | 1.075 | 0.845 | 3.251 | 0.846 |
| 2022-05-W1 | 1.846 | 0.840 | 1.800 | 0.847 | 1.846 | 0.840 | 1.846 | 0.840 | 3.638 | 0.848 |
| 2022-05-W2 | 2.091 | 0.845 | 2.214 | 0.850 | 2.311 | 0.844 | 2.311 | 0.844 | 4.004 | 0.853 |
| 2022-05-W3 | 1.845 | 0.847 | 1.930 | 0.851 | 1.543 | 0.845 | 1.543 | 0.845 | 2.961 | 0.849 |
| 2022-05-W4 | 4.874 | 0.842 | 3.782 | 0.844 | 4.474 | 0.827 | 4.474 | 0.827 | 5.895 | 0.840 |
| 2022-06-W1 | 3.934 | 0.864 | 3.857 | 0.864 | 3.783 | 0.864 | 4.525 | 0.856 | 5.874 | 0.862 |
| 2022-06-W2 | 3.004 | 0.859 | 3.048 | 0.860 | 3.097 | 0.859 | 4.373 | 0.848 | 5.464 | 0.859 |
| 2022-06-W3 | 3.067 | 0.822 | 2.481 | 0.824 | 2.741 | 0.825 | 3.958 | 0.818 | 5.589 | 0.824 |
| 2022-06-W4 | 1.308 | 0.832 | 1.305 | 0.841 | 1.682 | 0.841 | 2.215 | 0.834 | 4.436 | 0.842 |
| 2022-07-W1 | 2.377 | 0.833 | 2.261 | 0.834 | 2.345 | 0.833 | 2.366 | 0.834 | 2.412 | 0.834 |
| 2022-07-W2 | 1.357 | 0.819 | 1.776 | 0.818 | 1.715 | 0.818 | 1.707 | 0.819 | 1.725 | 0.818 |
| 2022-07-W3 | 1.300 | 0.839 | 1.454 | 0.838 | 0.711 | 0.839 | 0.805 | 0.841 | 0.760 | 0.838 |
| 2022-07-W4 | 1.091 | 0.846 | 0.803 | 0.844 | 1.863 | 0.847 | 1.912 | 0.849 | 1.897 | 0.848 |
| 2022-08-W1 | 3.544 | 0.837 | 3.544 | 0.837 | 3.662 | 0.842 | 3.346 | 0.840 | 3.392 | 0.840 |
| 2022-08-W2 | 4.238 | 0.829 | 3.759 | 0.829 | 3.868 | 0.831 | 4.300 | 0.827 | 4.122 | 0.828 |
| 2022-08-W3 | 0.981 | 0.822 | 0.981 | 0.822 | 1.024 | 0.831 | 1.544 | 0.830 | 1.486 | 0.830 |
| 2022-08-W4 | 1.446 | 0.835 | 2.032 | 0.829 | 1.997 | 0.834 | 2.838 | 0.831 | 2.699 | 0.832 |
| 2022-09-W1 | 1.072 | 0.847 | 1.072 | 0.847 | 1.072 | 0.847 | 1.072 | 0.847 | 3.742 | 0.871 |
| 2022-09-W2 | 7.595 | 0.851 | 6.994 | 0.837 | 6.994 | 0.837 | 6.994 | 0.837 | 9.542 | 0.855 |
| 2022-09-W3 | 3.528 | 0.846 | 3.528 | 0.846 | 2.930 | 0.834 | 2.930 | 0.834 | 5.138 | 0.851 |
| 2022-09-W4 | 2.062 | 0.850 | 1.904 | 0.852 | 2.420 | 0.838 | 2.420 | 0.838 | 2.337 | 0.855 |
| 2022-10-W1 | 1.764 | 0.860 | 1.764 | 0.860 | 1.764 | 0.860 | 1.186 | 0.850 | 1.764 | 0.860 |
| 2022-10-W2 | 5.727 | 0.837 | 5.987 | 0.835 | 5.987 | 0.835 | 5.529 | 0.833 | 5.987 | 0.835 |
| 2022-10-W3 | 1.220 | 0.844 | 1.056 | 0.844 | 1.667 | 0.841 | 1.928 | 0.832 | 1.667 | 0.841 |
| 2022-10-W4 | 1.395 | 0.860 | 1.277 | 0.862 | 1.801 | 0.854 | 1.775 | 0.843 | 1.801 | 0.854 |
| 2022-11-W1 | 4.796 | 0.829 | 4.941 | 0.828 | 4.717 | 0.829 | 4.850 | 0.830 | 14.026 | 0.800 |
| 2022-11-W2 | 2.047 | 0.824 | 7.617 | 0.821 | 7.109 | 0.821 | 7.098 | 0.821 | 26.150 | 0.798 |
| 2022-11-W3 | 2.728 | 0.859 | 2.166 | 0.859 | 10.641 | 0.856 | 10.582 | 0.859 | 30.526 | 0.837 |
| 2022-11-W4 | 3.530 | 0.857 | 2.246 | 0.857 | 7.686 | 0.858 | 7.626 | 0.857 | 27.864 | 0.831 |
| 2022-12-W1 | 2.152 | 0.867 | 1.437 | 0.865 | 1.551 | 0.866 | 8.047 | 0.857 | 25.628 | 0.847 |
| 2022-12-W2 | 2.638 | 0.821 | 0.865 | 0.841 | 1.035 | 0.842 | 8.181 | 0.832 | 26.030 | 0.817 |
| 2022-12-W3 | 0.696 | 0.856 | 0.915 | 0.845 | 1.161 | 0.856 | 9.478 | 0.854 | 27.851 | 0.831 |
| 2022-12-W4 | 0.887 | 0.849 | 0.907 | 0.850 | 0.641 | 0.861 | 7.692 | 0.852 | 24.203 | 0.817 |
| 2023-01-W1 | 3.650 | 0.819 | 3.550 | 0.819 | 3.687 | 0.820 | 3.000 | 0.823 | 3.731 | 0.821 |
| 2023-01-W2 | 3.859 | 0.852 | 4.973 | 0.858 | 5.096 | 0.859 | 4.135 | 0.857 | 5.120 | 0.858 |
| 2023-01-W3 | 3.126 | 0.857 | 3.126 | 0.857 | 4.444 | 0.867 | 3.915 | 0.863 | 4.476 | 0.868 |
| 2023-01-W4 | 2.854 | 0.856 | 2.718 | 0.857 | 3.497 | 0.869 | 2.994 | 0.861 | 3.584 | 0.870 |
| 2023-02-W1 | 2.259 | 0.854 | 2.259 | 0.854 | 2.259 | 0.854 | 3.090 | 0.854 | 3.755 | 0.861 |
| 2023-02-W2 | 2.509 | 0.851 | 2.356 | 0.851 | 2.356 | 0.851 | 1.445 | 0.854 | 0.977 | 0.857 |
| 2023-02-W3 | 1.327 | 0.851 | 1.327 | 0.851 | 1.579 | 0.845 | 1.663 | 0.853 | 2.754 | 0.856 |
| 2023-02-W4 | 1.715 | 0.860 | 1.093 | 0.863 | 1.663 | 0.864 | 1.343 | 0.860 | 2.149 | 0.862 |
| 2023-03-W1 | 1.195 | 0.846 | 1.049 | 0.847 | 1.043 | 0.847 | 1.277 | 0.848 | 1.390 | 0.847 |
| 2023-03-W2 | 3.438 | 0.846 | 3.184 | 0.846 | 3.258 | 0.846 | 3.106 | 0.847 | 2.492 | 0.842 |
| 2023-03-W3 | 2.224 | 0.857 | 2.097 | 0.855 | 2.106 | 0.853 | 1.971 | 0.854 | 1.309 | 0.855 |
| 2023-03-W4 | 3.560 | 0.829 | 3.413 | 0.827 | 3.316 | 0.827 | 3.556 | 0.828 | 4.134 | 0.828 |
| 2023-04-W1 | 0.283 | 0.854 | 0.225 | 0.851 | 0.225 | 0.851 | 0.261 | 0.851 | 0.225 | 0.851 |
| 2023-04-W2 | 1.766 | 0.828 | 1.598 | 0.848 | 1.598 | 0.848 | 0.823 | 0.841 | 1.598 | 0.848 |
| 2023-04-W3 | 0.072 | 0.847 | 0.072 | 0.847 | 0.217 | 0.848 | 0.314 | 0.851 | 0.217 | 0.848 |
| 2023-04-W4 | 0.476 | 0.839 | 1.197 | 0.847 | 1.448 | 0.847 | 1.255 | 0.854 | 1.448 | 0.847 |
| 2023-05-W1 | 2.355 | 0.834 | 2.355 | 0.834 | 2.355 | 0.834 | 2.355 | 0.834 | 2.844 | 0.837 |
| 2023-05-W2 | 0.768 | 0.827 | 0.069 | 0.833 | 0.069 | 0.833 | 0.069 | 0.833 | 0.793 | 0.835 |
| 2023-05-W3 | 1.574 | 0.837 | 1.574 | 0.837 | 1.735 | 0.835 | 1.735 | 0.835 | 0.898 | 0.841 |
| 2023-05-W4 | 4.144 | 0.828 | 3.981 | 0.822 | 4.020 | 0.824 | 4.020 | 0.824 | 3.496 | 0.827 |
| 2023-06-W1 | 5.101 | 0.845 | 5.101 | 0.845 | 5.101 | 0.845 | 6.016 | 0.842 | 5.267 | 0.846 |
| 2023-06-W2 | 3.927 | 0.841 | 4.302 | 0.847 | 4.302 | 0.847 | 4.880 | 0.841 | 4.124 | 0.848 |
| 2023-06-W3 | 3.800 | 0.853 | 3.800 | 0.853 | 4.811 | 0.868 | 5.559 | 0.863 | 4.871 | 0.869 |
| 2023-06-W4 | 2.221 | 0.818 | 2.205 | 0.820 | 2.621 | 0.835 | 3.458 | 0.834 | 2.634 | 0.832 |
| 2023-07-W1 | 3.024 | 0.842 | 3.024 | 0.842 | 3.024 | 0.842 | 3.024 | 0.842 | 3.024 | 0.842 |
| 2023-07-W2 | 2.995 | 0.861 | 1.881 | 0.844 | 1.881 | 0.844 | 1.881 | 0.844 | 1.881 | 0.844 |
| 2023-07-W3 | 0.063 | 0.849 | 0.063 | 0.849 | 0.792 | 0.822 | 0.792 | 0.822 | 0.792 | 0.822 |
| 2023-07-W4 | 1.164 | 0.856 | 1.443 | 0.856 | 1.471 | 0.844 | 1.471 | 0.844 | 1.471 | 0.844 |
| 2023-08-W1 | 3.276 | 0.832 | 3.276 | 0.832 | 3.276 | 0.832 | 1.791 | 0.828 | 1.791 | 0.828 |
| 2023-08-W2 | 3.681 | 0.847 | 3.292 | 0.846 | 3.292 | 0.846 | 3.034 | 0.832 | 3.034 | 0.832 |
| 2023-08-W3 | 1.662 | 0.845 | 1.662 | 0.845 | 1.608 | 0.843 | 1.364 | 0.814 | 1.364 | 0.814 |
| 2023-08-W4 | 3.271 | 0.851 | 3.162 | 0.850 | 3.244 | 0.845 | 2.960 | 0.840 | 2.960 | 0.840 |
| 2023-09-W1 | 2.403 | 0.851 | 2.403 | 0.851 | 2.403 | 0.851 | 2.403 | 0.851 | 2.791 | 0.816 |
| 2023-09-W2 | 5.453 | 0.828 | 5.414 | 0.842 | 5.414 | 0.842 | 5.414 | 0.842 | 5.088 | 0.828 |
| 2023-09-W3 | 1.593 | 0.852 | 1.593 | 0.852 | 1.597 | 0.857 | 1.597 | 0.857 | 1.957 | 0.835 |
| 2023-09-W4 | 2.137 | 0.829 | 3.291 | 0.842 | 3.216 | 0.843 | 3.216 | 0.843 | 3.512 | 0.830 |
| 2023-10-W1 | 1.092 | 0.856 | 1.224 | 0.855 | 1.079 | 0.857 | 1.051 | 0.849 | 1.153 | 0.855 |
| 2023-10-W2 | 3.412 | 0.827 | 3.306 | 0.827 | 3.521 | 0.828 | 3.462 | 0.820 | 3.321 | 0.828 |
| 2023-10-W3 | 1.779 | 0.864 | 2.592 | 0.855 | 1.797 | 0.865 | 1.501 | 0.855 | 1.655 | 0.864 |
| 2023-10-W4 | 1.674 | 0.863 | 1.173 | 0.866 | 1.244 | 0.872 | 1.907 | 0.868 | 1.174 | 0.872 |
| 2023-11-W1 | 2.526 | 0.835 | 2.526 | 0.835 | 2.526 | 0.835 | 2.526 | 0.835 | 1.713 | 0.840 |
| 2023-11-W2 | 2.102 | 0.850 | 2.107 | 0.850 | 2.107 | 0.850 | 2.107 | 0.850 | 0.737 | 0.860 |
| 2023-11-W3 | 0.371 | 0.820 | 0.289 | 0.820 | 0.599 | 0.808 | 0.599 | 0.808 | 0.058 | 0.819 |
| 2023-11-W4 | 0.486 | 0.845 | 0.694 | 0.846 | 1.366 | 0.838 | 1.366 | 0.838 | 0.734 | 0.845 |
| 2023-12-W1 | 1.745 | 0.829 | 1.745 | 0.829 | 1.466 | 0.844 | 0.934 | 0.835 | 1.212 | 0.850 |
| 2023-12-W2 | 1.201 | 0.847 | 1.424 | 0.842 | 1.242 | 0.848 | 2.013 | 0.844 | 1.126 | 0.848 |
| 2023-12-W3 | 1.882 | 0.823 | 1.833 | 0.823 | 1.706 | 0.823 | 1.042 | 0.809 | 1.524 | 0.820 |
| 2023-12-W4 | 1.785 | 0.824 | 1.831 | 0.825 | 1.951 | 0.825 | 1.113 | 0.813 | 1.680 | 0.820 |
| 2024-01-W1 | 0.942 | 0.844 | 0.942 | 0.844 | 0.942 | 0.844 | 0.942 | 0.844 | 0.942 | 0.844 |
| 2024-01-W2 | 1.373 | 0.820 | 1.651 | 0.824 | 1.651 | 0.824 | 1.651 | 0.824 | 1.651 | 0.824 |
| 2024-01-W3 | 2.967 | 0.807 | 2.967 | 0.807 | 2.822 | 0.825 | 2.822 | 0.825 | 2.822 | 0.825 |
| 2024-01-W4 | 1.455 | 0.829 | 1.571 | 0.832 | 1.443 | 0.852 | 1.443 | 0.852 | 1.443 | 0.852 |
| 2024-02-W1 | 1.059 | 0.834 | 1.059 | 0.834 | 1.088 | 0.841 | 1.152 | 0.834 | 1.152 | 0.834 |
| 2024-02-W2 | 1.275 | 0.823 | 1.255 | 0.832 | 1.369 | 0.843 | 0.963 | 0.836 | 0.963 | 0.836 |
| 2024-02-W3 | 1.773 | 0.836 | 1.773 | 0.836 | 0.912 | 0.860 | 0.642 | 0.850 | 0.642 | 0.850 |
| 2024-02-W4 | 2.976 | 0.854 | 3.786 | 0.843 | 2.690 | 0.858 | 2.119 | 0.854 | 2.119 | 0.854 |
| 2024-03-W1 | 1.881 | 0.843 | 1.881 | 0.843 | 1.881 | 0.843 | 1.881 | 0.843 | 1.822 | 0.850 |
| 2024-03-W2 | 2.761 | 0.801 | 2.919 | 0.814 | 2.919 | 0.814 | 2.919 | 0.814 | 3.140 | 0.818 |
| 2024-03-W3 | 6.225 | 0.849 | 6.208 | 0.849 | 6.074 | 0.838 | 6.074 | 0.838 | 6.309 | 0.845 |
| 2024-03-W4 | 2.313 | 0.833 | 2.076 | 0.856 | 2.259 | 0.842 | 2.259 | 0.842 | 2.259 | 0.847 |
| 2024-04-W1 | 1.246 | 0.796 | 1.246 | 0.796 | 1.246 | 0.796 | 1.181 | 0.803 | 1.246 | 0.796 |
| 2024-04-W2 | 0.540 | 0.829 | 0.570 | 0.828 | 0.570 | 0.828 | 0.717 | 0.833 | 0.570 | 0.828 |
| 2024-04-W3 | 0.908 | 0.832 | 0.908 | 0.832 | 0.739 | 0.834 | 0.326 | 0.841 | 0.739 | 0.834 |
| 2024-04-W4 | 0.764 | 0.861 | 0.444 | 0.846 | 0.628 | 0.843 | 0.766 | 0.847 | 0.628 | 0.843 |
| 2024-05-W1 | 0.854 | 0.860 | 0.981 | 0.860 | 0.985 | 0.860 | 0.794 | 0.860 | 1.132 | 0.837 |
| 2024-05-W2 | 0.552 | 0.847 | 0.026 | 0.859 | 0.066 | 0.860 | 0.137 | 0.860 | 0.314 | 0.844 |
| 2024-05-W3 | 2.122 | 0.862 | 2.196 | 0.861 | 2.013 | 0.859 | 1.917 | 0.859 | 1.763 | 0.829 |
| 2024-05-W4 | 1.823 | 0.864 | 1.839 | 0.862 | 2.009 | 0.869 | 1.931 | 0.869 | 2.193 | 0.856 |
| 2024-06-W1 | 1.953 | 0.838 | 1.384 | 0.853 | 1.953 | 0.838 | 1.455 | 0.851 | 1.838 | 0.830 |
| 2024-06-W2 | 0.989 | 0.851 | 1.063 | 0.850 | 1.401 | 0.845 | 1.182 | 0.854 | 1.465 | 0.833 |
| 2024-06-W3 | 1.636 | 0.858 | 1.636 | 0.858 | 1.631 | 0.859 | 1.324 | 0.868 | 1.497 | 0.855 |
| 2024-06-W4 | 2.473 | 0.849 | 2.688 | 0.833 | 2.138 | 0.841 | 2.408 | 0.852 | 2.655 | 0.828 |

Table S5: Primary outcomes for five most frequently retraining models

|  | **Half-yearly** | | **Yearly** | | **Biennial** | | **Triennial** | | **Static** | |
| --- | --- | --- | --- | --- | --- | --- | --- | --- | --- | --- |
| **Date** | **AMDBE** | **AUROC** | **AMDBE** | **AUROC** | **AMDBE** | **AUROC** | **AMDBE** | **AUROC** | **AMDBE** | **AUROC** |
| 2019-07-W1 | 2.639 | 0.857 | 2.238 | 0.852 | 2.601 | 0.856 | 2.720 | 0.857 | 3.483 | 0.852 |
| 2019-07-W2 | 3.759 | 0.866 | 3.811 | 0.863 | 3.751 | 0.867 | 3.822 | 0.866 | 3.811 | 0.863 |
| 2019-07-W3 | 1.726 | 0.871 | 1.138 | 0.863 | 1.727 | 0.870 | 1.615 | 0.871 | 1.138 | 0.863 |
| 2019-07-W4 | 1.231 | 0.851 | 1.138 | 0.853 | 1.197 | 0.852 | 1.221 | 0.851 | 1.138 | 0.853 |
| 2019-08-W1 | 2.935 | 0.858 | 3.262 | 0.855 | 2.970 | 0.858 | 3.054 | 0.857 | 3.262 | 0.855 |
| 2019-08-W2 | 3.924 | 0.854 | 3.100 | 0.856 | 3.914 | 0.855 | 3.906 | 0.855 | 3.100 | 0.856 |
| 2019-08-W3 | 2.723 | 0.872 | 3.293 | 0.868 | 2.746 | 0.873 | 2.784 | 0.872 | 3.293 | 0.868 |
| 2019-08-W4 | 2.224 | 0.858 | 2.701 | 0.856 | 2.266 | 0.858 | 2.241 | 0.858 | 2.701 | 0.856 |
| 2019-09-W1 | 4.458 | 0.860 | 5.754 | 0.859 | 4.498 | 0.861 | 4.471 | 0.860 | 5.754 | 0.859 |
| 2019-09-W2 | 1.339 | 0.851 | 1.894 | 0.843 | 1.329 | 0.851 | 1.417 | 0.851 | 1.894 | 0.843 |
| 2019-09-W3 | 4.387 | 0.866 | 4.850 | 0.863 | 4.385 | 0.866 | 4.366 | 0.865 | 4.850 | 0.863 |
| 2019-09-W4 | 2.488 | 0.854 | 2.850 | 0.850 | 2.513 | 0.854 | 2.526 | 0.854 | 2.850 | 0.850 |
| 2019-10-W1 | 1.352 | 0.854 | 1.263 | 0.845 | 1.402 | 0.854 | 1.361 | 0.855 | 1.263 | 0.845 |
| 2019-10-W2 | 4.714 | 0.835 | 5.213 | 0.831 | 4.771 | 0.835 | 4.777 | 0.835 | 5.213 | 0.831 |
| 2019-10-W3 | 3.096 | 0.871 | 3.527 | 0.864 | 3.156 | 0.872 | 3.163 | 0.871 | 3.527 | 0.864 |
| 2019-10-W4 | 1.829 | 0.867 | 1.368 | 0.862 | 1.744 | 0.867 | 1.763 | 0.866 | 1.368 | 0.862 |
| 2019-11-W1 | 4.515 | 0.862 | 5.073 | 0.856 | 4.601 | 0.862 | 4.538 | 0.861 | 5.073 | 0.856 |
| 2019-11-W2 | 1.647 | 0.867 | 1.833 | 0.860 | 1.652 | 0.866 | 1.692 | 0.866 | 1.833 | 0.860 |
| 2019-11-W3 | 1.315 | 0.864 | 1.149 | 0.858 | 1.224 | 0.864 | 1.268 | 0.864 | 1.149 | 0.858 |
| 2019-11-W4 | 1.735 | 0.854 | 1.816 | 0.853 | 1.742 | 0.855 | 1.732 | 0.855 | 1.816 | 0.853 |
| 2019-12-W1 | 1.410 | 0.871 | 1.627 | 0.869 | 1.475 | 0.871 | 1.448 | 0.871 | 1.627 | 0.869 |
| 2019-12-W2 | 1.502 | 0.835 | 1.780 | 0.829 | 1.561 | 0.834 | 1.475 | 0.834 | 1.780 | 0.829 |
| 2019-12-W3 | 1.238 | 0.839 | 1.321 | 0.838 | 1.242 | 0.839 | 1.308 | 0.839 | 1.321 | 0.838 |
| 2019-12-W4 | 2.414 | 0.811 | 2.279 | 0.806 | 2.444 | 0.810 | 2.394 | 0.810 | 2.279 | 0.806 |
| 2020-01-W1 | 2.095 | 0.842 | 1.768 | 0.844 | 1.876 | 0.847 | 1.854 | 0.848 | 1.768 | 0.844 |
| 2020-01-W2 | 0.894 | 0.839 | 1.232 | 0.852 | 0.909 | 0.856 | 0.943 | 0.855 | 1.232 | 0.852 |
| 2020-01-W3 | 4.285 | 0.834 | 4.649 | 0.836 | 4.468 | 0.842 | 4.537 | 0.843 | 4.649 | 0.836 |
| 2020-01-W4 | 1.186 | 0.842 | 1.576 | 0.852 | 1.427 | 0.851 | 1.448 | 0.851 | 1.576 | 0.852 |
| 2020-02-W1 | 2.080 | 0.860 | 2.915 | 0.860 | 2.850 | 0.865 | 2.859 | 0.864 | 2.915 | 0.860 |
| 2020-02-W2 | 1.472 | 0.859 | 2.141 | 0.865 | 2.000 | 0.867 | 1.976 | 0.867 | 2.141 | 0.865 |
| 2020-02-W3 | 3.774 | 0.862 | 4.563 | 0.861 | 4.081 | 0.867 | 4.101 | 0.866 | 4.563 | 0.861 |
| 2020-02-W4 | 2.561 | 0.848 | 1.354 | 0.849 | 1.798 | 0.851 | 1.781 | 0.850 | 1.354 | 0.849 |
| 2020-03-W1 | 2.292 | 0.846 | 2.713 | 0.855 | 2.634 | 0.857 | 2.602 | 0.857 | 2.713 | 0.855 |
| 2020-03-W2 | 1.380 | 0.852 | 2.419 | 0.851 | 2.710 | 0.856 | 2.684 | 0.855 | 2.419 | 0.851 |
| 2020-03-W3 | 3.360 | 0.829 | 3.519 | 0.835 | 3.135 | 0.843 | 3.264 | 0.842 | 3.519 | 0.835 |
| 2020-03-W4 | 0.657 | 0.840 | 0.693 | 0.842 | 0.638 | 0.848 | 0.649 | 0.849 | 0.693 | 0.842 |
| 2020-04-W1 | 1.695 | 0.809 | 1.533 | 0.826 | 0.961 | 0.828 | 1.011 | 0.829 | 1.533 | 0.826 |
| 2020-04-W2 | 3.041 | 0.823 | 2.825 | 0.832 | 2.618 | 0.838 | 2.661 | 0.838 | 2.825 | 0.832 |
| 2020-04-W3 | 2.336 | 0.814 | 2.042 | 0.820 | 1.891 | 0.826 | 1.967 | 0.825 | 2.042 | 0.820 |
| 2020-04-W4 | 2.926 | 0.824 | 3.217 | 0.832 | 2.948 | 0.832 | 3.023 | 0.833 | 3.217 | 0.832 |
| 2020-05-W1 | 2.302 | 0.815 | 2.824 | 0.821 | 2.479 | 0.827 | 2.527 | 0.826 | 2.824 | 0.821 |
| 2020-05-W2 | 3.173 | 0.815 | 3.127 | 0.820 | 2.589 | 0.827 | 2.619 | 0.828 | 3.127 | 0.820 |
| 2020-05-W3 | 1.494 | 0.827 | 1.661 | 0.842 | 1.330 | 0.846 | 1.330 | 0.845 | 1.661 | 0.842 |
| 2020-05-W4 | 1.796 | 0.848 | 2.206 | 0.843 | 1.843 | 0.851 | 1.867 | 0.851 | 2.206 | 0.843 |
| 2020-06-W1 | 1.790 | 0.838 | 1.395 | 0.838 | 1.871 | 0.836 | 1.815 | 0.837 | 1.395 | 0.838 |
| 2020-06-W2 | 2.021 | 0.855 | 1.937 | 0.854 | 1.980 | 0.858 | 2.031 | 0.858 | 1.937 | 0.854 |
| 2020-06-W3 | 0.934 | 0.841 | 0.847 | 0.854 | 0.622 | 0.856 | 0.590 | 0.857 | 0.847 | 0.854 |
| 2020-06-W4 | 4.821 | 0.834 | 4.465 | 0.840 | 3.979 | 0.839 | 4.010 | 0.838 | 4.465 | 0.840 |
| 2020-07-W1 | 1.047 | 0.848 | 0.885 | 0.850 | 1.095 | 0.850 | 1.130 | 0.851 | 1.118 | 0.850 |
| 2020-07-W2 | 3.653 | 0.845 | 3.616 | 0.848 | 3.699 | 0.847 | 3.783 | 0.847 | 4.071 | 0.849 |
| 2020-07-W3 | 1.086 | 0.852 | 1.270 | 0.851 | 0.841 | 0.851 | 0.885 | 0.851 | 0.570 | 0.855 |
| 2020-07-W4 | 0.992 | 0.843 | 0.938 | 0.842 | 1.222 | 0.837 | 1.254 | 0.837 | 1.119 | 0.836 |
| 2020-08-W1 | 1.792 | 0.848 | 1.588 | 0.851 | 1.715 | 0.846 | 1.767 | 0.846 | 2.028 | 0.845 |
| 2020-08-W2 | 2.436 | 0.841 | 2.325 | 0.838 | 2.243 | 0.840 | 2.318 | 0.839 | 2.680 | 0.840 |
| 2020-08-W3 | 2.535 | 0.861 | 2.484 | 0.861 | 3.051 | 0.861 | 3.087 | 0.860 | 3.337 | 0.860 |
| 2020-08-W4 | 12.785 | 0.843 | 12.945 | 0.840 | 12.415 | 0.839 | 12.556 | 0.839 | 12.586 | 0.836 |
| 2020-09-W1 | 1.773 | 0.856 | 1.972 | 0.854 | 2.016 | 0.852 | 2.216 | 0.852 | 1.863 | 0.853 |
| 2020-09-W2 | 2.551 | 0.849 | 2.629 | 0.850 | 2.445 | 0.849 | 2.388 | 0.849 | 2.809 | 0.840 |
| 2020-09-W3 | 2.224 | 0.873 | 2.255 | 0.872 | 2.336 | 0.865 | 2.390 | 0.865 | 2.095 | 0.862 |
| 2020-09-W4 | 1.485 | 0.852 | 1.398 | 0.849 | 1.645 | 0.841 | 1.615 | 0.842 | 1.895 | 0.833 |
| 2020-10-W1 | 4.631 | 0.819 | 4.498 | 0.821 | 4.206 | 0.827 | 4.246 | 0.826 | 4.182 | 0.819 |
| 2020-10-W2 | 2.301 | 0.844 | 2.385 | 0.842 | 2.056 | 0.836 | 2.041 | 0.836 | 1.740 | 0.821 |
| 2020-10-W3 | 3.120 | 0.862 | 3.160 | 0.858 | 2.964 | 0.857 | 2.998 | 0.857 | 2.854 | 0.853 |
| 2020-10-W4 | 3.197 | 0.827 | 3.083 | 0.828 | 2.966 | 0.824 | 2.940 | 0.825 | 2.925 | 0.818 |
| 2020-11-W1 | 3.214 | 0.852 | 3.220 | 0.849 | 2.984 | 0.844 | 3.015 | 0.843 | 2.798 | 0.841 |
| 2020-11-W2 | 0.856 | 0.833 | 0.752 | 0.831 | 1.285 | 0.830 | 1.338 | 0.829 | 1.714 | 0.826 |
| 2020-11-W3 | 6.876 | 0.858 | 6.818 | 0.856 | 6.538 | 0.852 | 6.564 | 0.852 | 6.290 | 0.847 |
| 2020-11-W4 | 2.910 | 0.829 | 3.122 | 0.827 | 2.738 | 0.820 | 2.753 | 0.819 | 2.239 | 0.814 |
| 2020-12-W1 | 4.566 | 0.844 | 4.347 | 0.842 | 4.404 | 0.843 | 4.349 | 0.842 | 3.914 | 0.831 |
| 2020-12-W2 | 4.370 | 0.842 | 4.186 | 0.842 | 4.059 | 0.839 | 3.957 | 0.838 | 3.546 | 0.837 |
| 2020-12-W3 | 4.099 | 0.825 | 3.961 | 0.823 | 3.611 | 0.823 | 3.497 | 0.821 | 3.079 | 0.818 |
| 2020-12-W4 | 5.102 | 0.849 | 5.007 | 0.849 | 4.348 | 0.845 | 4.196 | 0.845 | 3.837 | 0.841 |
| 2021-01-W1 | 2.735 | 0.862 | 2.555 | 0.853 | 2.239 | 0.850 | 2.198 | 0.849 | 1.810 | 0.842 |
| 2021-01-W2 | 1.412 | 0.846 | 1.218 | 0.841 | 1.137 | 0.841 | 1.130 | 0.841 | 1.268 | 0.838 |
| 2021-01-W3 | 4.278 | 0.858 | 4.327 | 0.857 | 4.815 | 0.857 | 4.872 | 0.856 | 5.082 | 0.847 |
| 2021-01-W4 | 4.134 | 0.836 | 4.208 | 0.831 | 4.035 | 0.823 | 4.102 | 0.823 | 4.355 | 0.815 |
| 2021-02-W1 | 1.318 | 0.852 | 1.331 | 0.854 | 1.893 | 0.849 | 1.851 | 0.849 | 2.237 | 0.838 |
| 2021-02-W2 | 1.680 | 0.850 | 2.106 | 0.851 | 2.168 | 0.844 | 2.219 | 0.843 | 1.566 | 0.839 |
| 2021-02-W3 | 2.270 | 0.863 | 2.636 | 0.861 | 2.377 | 0.857 | 2.439 | 0.857 | 2.172 | 0.855 |
| 2021-02-W4 | 2.141 | 0.834 | 1.892 | 0.832 | 2.174 | 0.826 | 2.207 | 0.825 | 2.874 | 0.820 |
| 2021-03-W1 | 1.803 | 0.874 | 1.876 | 0.869 | 1.454 | 0.863 | 1.566 | 0.864 | 1.286 | 0.857 |
| 2021-03-W2 | 2.796 | 0.849 | 3.241 | 0.844 | 2.249 | 0.846 | 2.429 | 0.845 | 2.053 | 0.843 |
| 2021-03-W3 | 2.057 | 0.851 | 1.582 | 0.850 | 2.008 | 0.842 | 2.026 | 0.841 | 2.514 | 0.828 |
| 2021-03-W4 | 6.732 | 0.863 | 6.924 | 0.863 | 6.566 | 0.861 | 6.602 | 0.861 | 5.851 | 0.854 |
| 2021-04-W1 | 2.632 | 0.845 | 2.220 | 0.845 | 2.282 | 0.842 | 2.282 | 0.842 | 2.389 | 0.841 |
| 2021-04-W2 | 0.842 | 0.852 | 1.096 | 0.852 | 0.805 | 0.852 | 0.892 | 0.852 | 0.750 | 0.849 |
| 2021-04-W3 | 2.010 | 0.862 | 2.661 | 0.863 | 2.569 | 0.858 | 2.721 | 0.857 | 2.284 | 0.855 |
| 2021-04-W4 | 2.227 | 0.836 | 2.730 | 0.831 | 2.629 | 0.823 | 2.645 | 0.822 | 2.284 | 0.813 |
| 2021-05-W1 | 1.267 | 0.852 | 1.725 | 0.852 | 1.659 | 0.851 | 1.819 | 0.849 | 1.238 | 0.841 |
| 2021-05-W2 | 1.025 | 0.878 | 0.983 | 0.877 | 0.897 | 0.881 | 0.923 | 0.881 | 1.406 | 0.877 |
| 2021-05-W3 | 3.310 | 0.849 | 3.805 | 0.847 | 4.093 | 0.844 | 4.170 | 0.843 | 3.495 | 0.834 |
| 2021-05-W4 | 2.638 | 0.848 | 2.751 | 0.845 | 2.599 | 0.841 | 2.616 | 0.841 | 1.898 | 0.836 |
| 2021-06-W1 | 2.155 | 0.850 | 1.725 | 0.847 | 1.289 | 0.845 | 1.324 | 0.844 | 1.652 | 0.839 |
| 2021-06-W2 | 2.228 | 0.846 | 2.789 | 0.843 | 2.371 | 0.839 | 2.438 | 0.838 | 1.621 | 0.836 |
| 2021-06-W3 | 5.356 | 0.878 | 6.066 | 0.876 | 6.026 | 0.870 | 6.087 | 0.869 | 5.526 | 0.859 |
| 2021-06-W4 | 2.435 | 0.839 | 2.888 | 0.839 | 2.170 | 0.835 | 2.180 | 0.834 | 1.195 | 0.836 |
| 2021-07-W1 | 0.868 | 0.839 | 0.868 | 0.839 | 0.868 | 0.839 | 2.658 | 0.854 | 2.064 | 0.847 |
| 2021-07-W2 | 0.701 | 0.830 | 0.701 | 0.830 | 0.701 | 0.830 | 1.746 | 0.838 | 1.227 | 0.834 |
| 2021-07-W3 | 1.672 | 0.821 | 1.672 | 0.821 | 1.672 | 0.821 | 2.989 | 0.832 | 2.558 | 0.825 |
| 2021-07-W4 | 7.887 | 0.852 | 7.887 | 0.852 | 7.887 | 0.852 | 9.996 | 0.848 | 8.554 | 0.844 |
| 2021-08-W1 | 10.391 | 0.850 | 10.391 | 0.850 | 10.391 | 0.850 | 11.658 | 0.845 | 10.947 | 0.838 |
| 2021-08-W2 | 11.795 | 0.839 | 11.795 | 0.839 | 11.795 | 0.839 | 13.774 | 0.849 | 13.114 | 0.844 |
| 2021-08-W3 | 8.037 | 0.855 | 8.037 | 0.855 | 8.037 | 0.855 | 10.904 | 0.862 | 9.920 | 0.856 |
| 2021-08-W4 | 8.479 | 0.850 | 8.479 | 0.850 | 8.479 | 0.850 | 9.656 | 0.852 | 8.846 | 0.847 |
| 2021-09-W1 | 1.613 | 0.833 | 1.613 | 0.833 | 1.613 | 0.833 | 2.602 | 0.832 | 1.827 | 0.826 |
| 2021-09-W2 | 2.721 | 0.856 | 2.721 | 0.856 | 2.721 | 0.856 | 4.197 | 0.855 | 3.067 | 0.850 |
| 2021-09-W3 | 5.313 | 0.855 | 5.313 | 0.855 | 5.313 | 0.855 | 6.045 | 0.853 | 5.378 | 0.848 |
| 2021-09-W4 | 1.994 | 0.842 | 1.994 | 0.842 | 1.994 | 0.842 | 2.190 | 0.844 | 1.669 | 0.835 |
| 2021-10-W1 | 2.768 | 0.823 | 2.768 | 0.823 | 2.768 | 0.823 | 4.853 | 0.839 | 4.382 | 0.834 |
| 2021-10-W2 | 2.260 | 0.860 | 2.260 | 0.860 | 2.260 | 0.860 | 3.561 | 0.870 | 3.015 | 0.865 |
| 2021-10-W3 | 2.315 | 0.813 | 2.315 | 0.813 | 2.315 | 0.813 | 2.793 | 0.824 | 2.225 | 0.820 |
| 2021-10-W4 | 2.347 | 0.847 | 2.347 | 0.847 | 2.347 | 0.847 | 3.238 | 0.846 | 2.414 | 0.839 |
| 2021-11-W1 | 6.389 | 0.836 | 6.389 | 0.836 | 6.389 | 0.836 | 7.250 | 0.838 | 6.267 | 0.832 |
| 2021-11-W2 | 5.753 | 0.807 | 5.753 | 0.807 | 5.753 | 0.807 | 7.490 | 0.820 | 6.442 | 0.810 |
| 2021-11-W3 | 5.009 | 0.851 | 5.009 | 0.851 | 5.009 | 0.851 | 5.847 | 0.849 | 5.603 | 0.847 |
| 2021-11-W4 | 5.953 | 0.813 | 5.953 | 0.813 | 5.953 | 0.813 | 8.233 | 0.821 | 7.562 | 0.821 |
| 2021-12-W1 | 3.674 | 0.828 | 3.674 | 0.828 | 3.674 | 0.828 | 5.311 | 0.836 | 4.431 | 0.830 |
| 2021-12-W2 | 5.155 | 0.840 | 5.155 | 0.840 | 5.155 | 0.840 | 6.741 | 0.843 | 5.503 | 0.836 |
| 2021-12-W3 | 1.014 | 0.817 | 1.014 | 0.817 | 1.014 | 0.817 | 3.180 | 0.821 | 2.424 | 0.814 |
| 2021-12-W4 | 2.718 | 0.834 | 2.718 | 0.834 | 2.718 | 0.834 | 2.845 | 0.839 | 2.092 | 0.840 |
| 2022-01-W1 | 4.412 | 0.823 | 3.371 | 0.820 | 3.371 | 0.820 | 3.781 | 0.821 | 4.069 | 0.811 |
| 2022-01-W2 | 1.939 | 0.818 | 0.687 | 0.805 | 0.687 | 0.805 | 1.221 | 0.818 | 0.854 | 0.807 |
| 2022-01-W3 | 1.875 | 0.825 | 1.204 | 0.809 | 1.204 | 0.809 | 1.874 | 0.802 | 1.265 | 0.798 |
| 2022-01-W4 | 1.663 | 0.822 | 2.711 | 0.809 | 2.711 | 0.809 | 3.857 | 0.834 | 3.542 | 0.829 |
| 2022-02-W1 | 4.523 | 0.836 | 2.498 | 0.825 | 2.498 | 0.825 | 4.088 | 0.828 | 5.310 | 0.796 |
| 2022-02-W2 | 11.711 | 0.831 | 8.324 | 0.829 | 8.324 | 0.829 | 11.930 | 0.848 | 14.125 | 0.831 |
| 2022-02-W3 | 10.118 | 0.779 | 7.039 | 0.770 | 7.039 | 0.770 | 10.373 | 0.773 | 12.811 | 0.765 |
| 2022-02-W4 | 6.707 | 0.821 | 4.132 | 0.828 | 4.132 | 0.828 | 7.863 | 0.839 | 10.383 | 0.829 |
| 2022-03-W1 | 8.828 | 0.797 | 5.591 | 0.814 | 5.591 | 0.814 | 9.161 | 0.815 | 11.467 | 0.792 |
| 2022-03-W2 | 7.811 | 0.763 | 4.333 | 0.775 | 4.333 | 0.775 | 7.969 | 0.801 | 10.395 | 0.788 |
| 2022-03-W3 | 9.487 | 0.780 | 6.598 | 0.799 | 6.598 | 0.799 | 8.946 | 0.793 | 11.724 | 0.779 |
| 2022-03-W4 | 6.369 | 0.810 | 3.163 | 0.813 | 3.163 | 0.813 | 6.764 | 0.812 | 9.003 | 0.800 |
| 2022-04-W1 | 8.690 | 0.774 | 5.421 | 0.781 | 5.421 | 0.781 | 9.794 | 0.794 | 12.463 | 0.779 |
| 2022-04-W2 | 7.807 | 0.806 | 5.023 | 0.794 | 5.023 | 0.794 | 8.146 | 0.812 | 10.195 | 0.794 |
| 2022-04-W3 | 10.304 | 0.822 | 7.709 | 0.822 | 7.709 | 0.822 | 11.068 | 0.840 | 13.419 | 0.818 |
| 2022-04-W4 | 9.562 | 0.799 | 5.829 | 0.810 | 5.829 | 0.810 | 9.243 | 0.824 | 11.612 | 0.803 |
| 2022-05-W1 | 10.693 | 0.806 | 7.179 | 0.807 | 7.179 | 0.807 | 10.052 | 0.818 | 12.746 | 0.806 |
| 2022-05-W2 | 11.919 | 0.807 | 7.344 | 0.816 | 7.344 | 0.816 | 12.430 | 0.825 | 15.918 | 0.808 |
| 2022-05-W3 | 9.706 | 0.838 | 5.755 | 0.829 | 5.755 | 0.829 | 9.685 | 0.841 | 12.633 | 0.827 |
| 2022-05-W4 | 15.208 | 0.795 | 10.871 | 0.803 | 10.871 | 0.803 | 13.847 | 0.825 | 16.872 | 0.819 |
| 2022-06-W1 | 13.642 | 0.828 | 9.571 | 0.829 | 9.571 | 0.829 | 13.228 | 0.843 | 16.482 | 0.832 |
| 2022-06-W2 | 14.642 | 0.822 | 10.270 | 0.812 | 10.270 | 0.812 | 13.779 | 0.822 | 16.818 | 0.804 |
| 2022-06-W3 | 13.068 | 0.789 | 8.374 | 0.798 | 8.374 | 0.798 | 12.859 | 0.804 | 15.373 | 0.781 |
| 2022-06-W4 | 11.932 | 0.799 | 8.474 | 0.802 | 8.474 | 0.802 | 12.422 | 0.824 | 15.246 | 0.801 |
| 2022-07-W1 | 2.286 | 0.834 | 2.458 | 0.832 | 5.909 | 0.786 | 2.421 | 0.833 | 12.347 | 0.795 |
| 2022-07-W2 | 1.782 | 0.818 | 1.592 | 0.817 | 8.352 | 0.787 | 1.703 | 0.818 | 15.950 | 0.796 |
| 2022-07-W3 | 0.739 | 0.838 | 0.778 | 0.839 | 7.771 | 0.807 | 0.797 | 0.839 | 13.819 | 0.805 |
| 2022-07-W4 | 1.834 | 0.847 | 1.957 | 0.847 | 8.365 | 0.815 | 1.969 | 0.848 | 15.838 | 0.809 |
| 2022-08-W1 | 3.433 | 0.841 | 3.205 | 0.842 | 10.824 | 0.806 | 3.329 | 0.840 | 18.249 | 0.803 |
| 2022-08-W2 | 4.063 | 0.828 | 4.241 | 0.828 | 6.648 | 0.790 | 4.216 | 0.828 | 13.670 | 0.799 |
| 2022-08-W3 | 1.414 | 0.830 | 1.598 | 0.830 | 7.774 | 0.797 | 1.608 | 0.831 | 16.194 | 0.794 |
| 2022-08-W4 | 2.701 | 0.832 | 2.811 | 0.832 | 9.693 | 0.808 | 2.797 | 0.832 | 17.467 | 0.788 |
| 2022-09-W1 | 3.723 | 0.871 | 3.959 | 0.872 | 7.453 | 0.837 | 3.894 | 0.872 | 14.447 | 0.827 |
| 2022-09-W2 | 9.575 | 0.854 | 9.811 | 0.855 | 2.946 | 0.826 | 9.674 | 0.855 | 12.133 | 0.818 |
| 2022-09-W3 | 5.105 | 0.851 | 5.339 | 0.851 | 6.478 | 0.829 | 5.333 | 0.851 | 15.327 | 0.804 |
| 2022-09-W4 | 2.363 | 0.855 | 2.586 | 0.855 | 9.364 | 0.833 | 2.525 | 0.855 | 17.303 | 0.818 |
| 2022-10-W1 | 3.388 | 0.866 | 3.569 | 0.866 | 7.467 | 0.831 | 3.591 | 0.866 | 14.344 | 0.841 |
| 2022-10-W2 | 4.553 | 0.843 | 4.397 | 0.842 | 12.530 | 0.796 | 4.421 | 0.843 | 19.266 | 0.795 |
| 2022-10-W3 | 1.871 | 0.844 | 2.080 | 0.844 | 9.051 | 0.807 | 1.985 | 0.844 | 16.978 | 0.820 |
| 2022-10-W4 | 2.207 | 0.857 | 2.452 | 0.858 | 7.830 | 0.819 | 2.421 | 0.858 | 14.359 | 0.826 |
| 2022-11-W1 | 5.125 | 0.829 | 5.871 | 0.829 | 8.230 | 0.800 | 5.092 | 0.829 | 15.808 | 0.803 |
| 2022-11-W2 | 7.303 | 0.820 | 8.598 | 0.820 | 8.095 | 0.786 | 7.115 | 0.820 | 14.871 | 0.791 |
| 2022-11-W3 | 10.743 | 0.855 | 12.200 | 0.854 | 6.294 | 0.808 | 10.518 | 0.855 | 11.588 | 0.803 |
| 2022-11-W4 | 7.539 | 0.854 | 8.851 | 0.854 | 9.168 | 0.819 | 7.301 | 0.854 | 14.531 | 0.812 |
| 2022-12-W1 | 7.475 | 0.860 | 8.770 | 0.859 | 7.777 | 0.811 | 7.400 | 0.860 | 14.364 | 0.810 |
| 2022-12-W2 | 8.063 | 0.834 | 9.550 | 0.835 | 7.036 | 0.807 | 7.900 | 0.835 | 12.921 | 0.798 |
| 2022-12-W3 | 9.550 | 0.846 | 10.970 | 0.845 | 5.542 | 0.809 | 9.398 | 0.846 | 11.052 | 0.812 |
| 2022-12-W4 | 7.882 | 0.855 | 9.214 | 0.855 | 4.790 | 0.829 | 7.838 | 0.856 | 10.900 | 0.816 |
| 2023-01-W1 | 3.583 | 0.819 | 7.338 | 0.818 | 7.153 | 0.792 | 5.971 | 0.817 | 11.992 | 0.770 |
| 2023-01-W2 | 4.978 | 0.858 | 4.848 | 0.857 | 8.120 | 0.822 | 3.631 | 0.859 | 12.517 | 0.808 |
| 2023-01-W3 | 4.204 | 0.866 | 4.837 | 0.872 | 8.496 | 0.840 | 3.596 | 0.871 | 13.705 | 0.807 |
| 2023-01-W4 | 3.354 | 0.869 | 7.206 | 0.861 | 8.856 | 0.841 | 5.896 | 0.862 | 13.991 | 0.836 |
| 2023-02-W1 | 3.452 | 0.862 | 7.924 | 0.859 | 7.246 | 0.831 | 6.604 | 0.859 | 13.297 | 0.826 |
| 2023-02-W2 | 0.723 | 0.857 | 10.465 | 0.848 | 6.623 | 0.811 | 8.826 | 0.848 | 10.963 | 0.818 |
| 2023-02-W3 | 2.373 | 0.855 | 8.760 | 0.848 | 8.930 | 0.820 | 7.205 | 0.848 | 14.901 | 0.824 |
| 2023-02-W4 | 1.993 | 0.862 | 9.631 | 0.857 | 5.640 | 0.835 | 8.237 | 0.856 | 12.833 | 0.817 |
| 2023-03-W1 | 1.125 | 0.845 | 9.475 | 0.843 | 7.017 | 0.795 | 8.066 | 0.843 | 11.196 | 0.794 |
| 2023-03-W2 | 2.709 | 0.843 | 11.098 | 0.846 | 3.810 | 0.805 | 9.680 | 0.847 | 9.221 | 0.786 |
| 2023-03-W3 | 1.578 | 0.854 | 10.714 | 0.851 | 5.221 | 0.827 | 9.211 | 0.851 | 10.402 | 0.828 |
| 2023-03-W4 | 3.986 | 0.828 | 8.596 | 0.820 | 9.107 | 0.797 | 7.132 | 0.819 | 15.234 | 0.787 |
| 2023-04-W1 | 0.008 | 0.852 | 9.800 | 0.852 | 5.484 | 0.821 | 8.392 | 0.853 | 11.690 | 0.797 |
| 2023-04-W2 | 1.170 | 0.846 | 7.534 | 0.849 | 6.137 | 0.810 | 6.395 | 0.848 | 11.084 | 0.799 |
| 2023-04-W3 | 0.085 | 0.848 | 8.384 | 0.841 | 5.423 | 0.785 | 7.107 | 0.841 | 10.284 | 0.796 |
| 2023-04-W4 | 1.507 | 0.850 | 7.427 | 0.848 | 7.673 | 0.811 | 6.113 | 0.848 | 12.484 | 0.806 |
| 2023-05-W1 | 3.246 | 0.837 | 6.182 | 0.828 | 7.869 | 0.794 | 4.880 | 0.828 | 13.117 | 0.764 |
| 2023-05-W2 | 0.797 | 0.840 | 10.889 | 0.835 | 7.827 | 0.787 | 9.200 | 0.835 | 13.077 | 0.789 |
| 2023-05-W3 | 0.830 | 0.841 | 13.111 | 0.835 | 7.761 | 0.779 | 11.329 | 0.835 | 13.526 | 0.786 |
| 2023-05-W4 | 3.316 | 0.830 | 15.665 | 0.827 | 7.020 | 0.790 | 13.772 | 0.828 | 12.480 | 0.801 |
| 2023-06-W1 | 5.138 | 0.843 | 16.796 | 0.840 | 4.247 | 0.801 | 14.955 | 0.840 | 10.557 | 0.791 |
| 2023-06-W2 | 3.944 | 0.846 | 16.447 | 0.841 | 6.008 | 0.806 | 14.579 | 0.842 | 11.778 | 0.798 |
| 2023-06-W3 | 4.752 | 0.877 | 16.206 | 0.871 | 6.081 | 0.824 | 14.404 | 0.871 | 11.876 | 0.820 |
| 2023-06-W4 | 2.928 | 0.842 | 14.640 | 0.835 | 6.945 | 0.802 | 12.973 | 0.836 | 12.726 | 0.794 |
| 2023-07-W1 | 3.024 | 0.842 | 3.024 | 0.842 | 3.024 | 0.842 | 14.923 | 0.855 | 10.447 | 0.811 |
| 2023-07-W2 | 1.881 | 0.844 | 1.881 | 0.844 | 1.881 | 0.844 | 13.338 | 0.863 | 11.932 | 0.804 |
| 2023-07-W3 | 0.792 | 0.822 | 0.792 | 0.822 | 0.792 | 0.822 | 10.929 | 0.838 | 12.535 | 0.805 |
| 2023-07-W4 | 1.471 | 0.844 | 1.471 | 0.844 | 1.471 | 0.844 | 11.225 | 0.853 | 13.047 | 0.826 |
| 2023-08-W1 | 1.791 | 0.828 | 1.791 | 0.828 | 1.791 | 0.828 | 15.274 | 0.829 | 11.753 | 0.791 |
| 2023-08-W2 | 3.034 | 0.832 | 3.034 | 0.832 | 3.034 | 0.832 | 15.390 | 0.836 | 11.767 | 0.796 |
| 2023-08-W3 | 1.364 | 0.814 | 1.364 | 0.814 | 1.364 | 0.814 | 13.331 | 0.836 | 13.066 | 0.797 |
| 2023-08-W4 | 2.960 | 0.840 | 2.960 | 0.840 | 2.960 | 0.840 | 8.822 | 0.841 | 15.368 | 0.817 |
| 2023-09-W1 | 2.791 | 0.816 | 2.791 | 0.816 | 2.791 | 0.816 | 11.149 | 0.842 | 12.887 | 0.811 |
| 2023-09-W2 | 5.088 | 0.828 | 5.088 | 0.828 | 5.088 | 0.828 | 6.061 | 0.837 | 16.803 | 0.773 |
| 2023-09-W3 | 1.957 | 0.835 | 1.957 | 0.835 | 1.957 | 0.835 | 9.732 | 0.860 | 11.874 | 0.812 |
| 2023-09-W4 | 3.512 | 0.830 | 3.512 | 0.830 | 3.512 | 0.830 | 9.961 | 0.845 | 12.692 | 0.798 |
| 2023-10-W1 | 2.194 | 0.831 | 2.194 | 0.831 | 2.194 | 0.831 | 8.781 | 0.849 | 11.596 | 0.812 |
| 2023-10-W2 | 5.128 | 0.794 | 5.128 | 0.794 | 5.128 | 0.794 | 7.344 | 0.826 | 15.252 | 0.779 |
| 2023-10-W3 | 2.489 | 0.841 | 2.489 | 0.841 | 2.489 | 0.841 | 9.433 | 0.862 | 11.519 | 0.826 |
| 2023-10-W4 | 2.364 | 0.853 | 2.364 | 0.853 | 2.364 | 0.853 | 8.099 | 0.868 | 13.478 | 0.840 |
| 2023-11-W1 | 1.122 | 0.805 | 1.122 | 0.805 | 1.122 | 0.805 | 10.380 | 0.846 | 12.464 | 0.800 |
| 2023-11-W2 | 0.864 | 0.834 | 0.864 | 0.834 | 0.864 | 0.834 | 9.873 | 0.862 | 12.229 | 0.812 |
| 2023-11-W3 | 0.297 | 0.797 | 0.297 | 0.797 | 0.297 | 0.797 | 9.207 | 0.817 | 11.632 | 0.743 |
| 2023-11-W4 | 1.513 | 0.830 | 1.513 | 0.830 | 1.513 | 0.830 | 9.023 | 0.842 | 12.773 | 0.805 |
| 2023-12-W1 | 2.071 | 0.828 | 2.071 | 0.828 | 2.071 | 0.828 | 7.794 | 0.834 | 11.879 | 0.787 |
| 2023-12-W2 | 2.356 | 0.830 | 2.356 | 0.830 | 2.356 | 0.830 | 8.948 | 0.847 | 10.772 | 0.800 |
| 2023-12-W3 | 2.034 | 0.806 | 2.034 | 0.806 | 2.034 | 0.806 | 7.151 | 0.813 | 10.956 | 0.777 |
| 2023-12-W4 | 2.234 | 0.792 | 2.234 | 0.792 | 2.234 | 0.792 | 6.823 | 0.811 | 10.350 | 0.783 |
| 2024-01-W1 | 0.942 | 0.844 | 1.187 | 0.813 | 1.187 | 0.813 | 6.923 | 0.843 | 9.285 | 0.804 |
| 2024-01-W2 | 1.651 | 0.824 | 2.449 | 0.808 | 2.449 | 0.808 | 8.436 | 0.821 | 10.444 | 0.770 |
| 2024-01-W3 | 2.822 | 0.825 | 3.338 | 0.803 | 3.338 | 0.803 | 5.437 | 0.828 | 11.099 | 0.774 |
| 2024-01-W4 | 1.443 | 0.852 | 1.636 | 0.834 | 1.636 | 0.834 | 7.638 | 0.858 | 10.376 | 0.804 |
| 2024-02-W1 | 1.152 | 0.834 | 1.463 | 0.801 | 1.463 | 0.801 | 7.612 | 0.829 | 11.084 | 0.777 |
| 2024-02-W2 | 0.963 | 0.836 | 0.262 | 0.822 | 0.262 | 0.822 | 9.952 | 0.839 | 10.720 | 0.800 |
| 2024-02-W3 | 0.642 | 0.850 | 0.055 | 0.837 | 0.055 | 0.837 | 9.794 | 0.857 | 11.701 | 0.806 |
| 2024-02-W4 | 2.119 | 0.854 | 1.776 | 0.843 | 1.776 | 0.843 | 11.302 | 0.851 | 8.978 | 0.805 |
| 2024-03-W1 | 1.822 | 0.850 | 1.542 | 0.831 | 1.542 | 0.831 | 7.330 | 0.854 | 12.397 | 0.805 |
| 2024-03-W2 | 3.140 | 0.818 | 2.203 | 0.795 | 2.203 | 0.795 | 12.450 | 0.824 | 8.577 | 0.772 |
| 2024-03-W3 | 6.309 | 0.845 | 5.725 | 0.827 | 5.725 | 0.827 | 14.147 | 0.848 | 4.633 | 0.795 |
| 2024-03-W4 | 2.259 | 0.847 | 2.266 | 0.819 | 2.266 | 0.819 | 9.748 | 0.849 | 10.131 | 0.825 |
| 2024-04-W1 | 0.729 | 0.812 | 1.447 | 0.795 | 1.447 | 0.795 | 9.918 | 0.815 | 10.568 | 0.769 |
| 2024-04-W2 | 1.007 | 0.839 | 0.479 | 0.817 | 0.479 | 0.817 | 9.204 | 0.835 | 8.635 | 0.789 |
| 2024-04-W3 | 0.273 | 0.850 | 1.649 | 0.817 | 1.649 | 0.817 | 7.590 | 0.844 | 10.275 | 0.801 |
| 2024-04-W4 | 0.894 | 0.854 | 0.749 | 0.830 | 0.749 | 0.830 | 9.558 | 0.850 | 10.061 | 0.797 |
| 2024-05-W1 | 1.195 | 0.848 | 0.398 | 0.832 | 0.398 | 0.832 | 9.987 | 0.854 | 10.573 | 0.820 |
| 2024-05-W2 | 0.362 | 0.855 | 0.166 | 0.836 | 0.166 | 0.836 | 9.878 | 0.854 | 12.353 | 0.818 |
| 2024-05-W3 | 1.970 | 0.837 | 1.411 | 0.832 | 1.411 | 0.832 | 10.466 | 0.845 | 11.133 | 0.796 |
| 2024-05-W4 | 2.048 | 0.866 | 1.818 | 0.835 | 1.818 | 0.835 | 10.032 | 0.867 | 11.970 | 0.816 |
| 2024-06-W1 | 1.685 | 0.840 | 1.235 | 0.819 | 1.235 | 0.819 | 9.838 | 0.848 | 10.146 | 0.801 |
| 2024-06-W2 | 1.511 | 0.849 | 1.386 | 0.839 | 1.386 | 0.839 | 10.024 | 0.852 | 11.220 | 0.802 |
| 2024-06-W3 | 1.282 | 0.859 | 1.095 | 0.850 | 1.095 | 0.850 | 11.280 | 0.862 | 12.645 | 0.824 |
| 2024-06-W4 | 1.895 | 0.843 | 3.039 | 0.826 | 3.039 | 0.826 | 9.801 | 0.845 | 15.716 | 0.803 |

Table S6: Primary outcomes for the five least frequently retrained models
