# Supplementary material for: Optimising Retraining Frequency for a Paediatric Emergency Department Admission Prediction Model: Development and Temporal Validation Using Real‐World Data
Source: Emerg Med Australas. 2026 May 6;38:e70271. doi: 10.1111/1742-6723.70271 (PMC13146150; doi:10.1111/1742-6723.70271)
Supplement: Supplementary file 2 — Appendix S2: emm70271‐sup‐0002‐Appendicx_S2.docx. [file EMM-38-0-s002.docx]

TRIPOD + AI

| **Section/Topic** | **Item** | **Development / Evaluation** | **Checklist item** | **Reported on Section/Paragraph** |
| --- | --- | --- | --- | --- |
| **Title** | 1 | D;E | Identify the study as developing or evaluating the performance of a multivariable prediction model, the target population, and the outcome to be predicted | Title |
| **Abstract** | 2 | D;E | See TRIPOD+AI for Abstracts checklist | Abstract |
| **Introduction** | | | | |
| Background | 3a | D;E | Explain the healthcare context (including whether diagnostic or prognostic) and rationale for developing or evaluating the prediction model, including references to existing models | Introduction: paragraph 1–4 |
|  | 3b | D;E | Describe the target population and the intended purpose of the prediction model in the context of the care pathway, including its intended users (e.g., healthcare professionals, patients, public) | Introduction: paragraph 3–5 |
|  | 3c | D;E | Describe any known health inequalities between sociodemographic groups | Introduction: paragraph 4 & Methods: Evaluation Metrics |
| Objectives | 4 | D;E | Specify the study objectives, including whether the study describes the development or validation of a prediction model (or both) | Introduction: last paragraph |
| **Methods** |  |  |  |  |
| Data | 5a | D;E | Describe the sources of data separately for the development and evaluation datasets, the rationale for using these data, and representativeness of the data | Methods: Study Design and Overview + Data Source and Cohort |
|  | 5b | D;E | Specify the dates of the collected participant data, including start and end of participant accrual; and, if applicable, end of follow-up | Methods: Data Source and Cohort (July 1, 2018 – June 30, 2024) |
| Participants | 6a | D;E | Specify key elements of the study setting including the number and location of centres | Methods: Data Source and Cohort (Perth Children’s Hospital) |
|  | 6b | D;E | Describe the eligibility criteria for study participants | Methods: Data Source and Cohort (exclusions n=477) |
|  | 6c | D;E | Give details of any treatments received, and how they were handled during model development or evaluation, if relevant | N/A (no interventions) |
| Data preparation | 7 | D;E | Describe any data pre-processing and quality checking, including whether this was similar across relevant sociodemographic groups | Methods: Data Source and Cohort + Appendix A |
| Outcome | 8a | D;E | Clearly define the outcome that is being predicted and the time horizon, including how and when assessed | Methods: Data Source and Cohort |
|  | 8b | D;E | If outcome assessment requires subjective interpretation, describe the qualifications and demographic characteristics of the outcome assessors | N/A (objective administrative outcome) |
|  | 8c | D;E | Report any actions to blind assessment of the outcome to be predicted | Strict temporal separation discussed throughout paper |
| Predictors | 9a | D | Describe the choice of initial predictors and any pre-selection before model building | Methods: Data Source and Cohort |
|  | 9b | D;E | Clearly define all predictors, including how and when they were measured | Methods: Data Source and Cohort (full list) |
|  | 9c | D;E | If predictor measurement requires subjective interpretation, describe the qualifications… | Appendix A (triage nurse free-text to a tuned BioClinicalBERT embedding model) |
| Sample size | 10 | D;E | Explain how the study size was arrived at and justify sufficiency | Methods: Data Source and Cohort & Results |
| Missing data | 11 | D;E | Describe how missing data were handled. Provide reasons for omitting any data | Methods: Data Source and Cohort |
| Analytical methods | 12a | D | Describe how the data were used in the analysis, including partitioning | Methods: Study Design and Overview + Appendix A |
|  | 12b | D | Depending on the type of model, describe how predictors were handled | Appendix A (embeddings, SMOTE, etc.) |
|  | 12c | D | Specify the type of model, rationale, all model-building steps, including any hyperparameter tuning, and method for internal validation | Methods: Study Design and Overview + Appendix A |
|  | 12d | D;E | Describe if and how any heterogeneity across clusters was handled | N/A (single centre) |
|  | 12e | D;E | Specify all measures and plots used to evaluate model performance | Methods: Evaluation Metrics & Appendix A Table S2 |
|  | 12f | E | Describe any model updating (recalibration) | Core objective of the study |
|  | 12g | E | For model evaluation, describe how the model predictions were calculated | Appendix A |
| Class imbalance | 13 | D;E | If class imbalance methods were used, state why and how | Appendix A (SMOTE for XGB + post-hoc calibration) |
| Fairness | 14 | D;E | Describe any approaches used to address model fairness and their rationale | Methods: Evaluation Metrics  Additional note: Socioeconomic and racial variables were not included, as the model was designed for aggregated bed forecasting rather than individual-level decision-making, to minimise the risk of inequitable downstream use. |
| Model output | 15 | D | Specify the output of the prediction model (probabilities, classification). Provide details and rationale for any classification thresholds | Methods: Evaluation Metrics (probabilistic, summed for daily bed forecast) |
| Training versus evaluation | 16 | D;E | Identify any differences between the development and evaluation data | Appendix A (strict temporal separation only) |
| Ethical approval | 17 | D;E | Name the institutional research board or ethics committee… | Methods: Study Design and Overview (final paragraph) |
| **Open Science** |  |  |  |  |
| Funding | 18a | D;E | Give the source of funding and the role of the funders | Acknowledgements |
| Conflicts of interest | 18b | D;E | Declare any conflicts of interest and financial disclosures for all authors | None declared |
| Protocol | 18c | D;E | Indicate where the study protocol can be accessed or state not prepared | No protocol published prior |
| Registration | 18d | D;E | Provide registration information or state not registered | Methods: registered with two ethics bodies |
| Data sharing | 18e | D;E | Provide details of the availability of the study data | Patient data is not available as per our ethics agreement |
| Code sharing | 18f | D;E | Provide details of the availability of the analytical code | Methods: code may be available upon reasonable request |
| **Patient & Public Involvement** | 19 | D;E | Provide details of any patient and public involvement or state no involvement | No involvement |
| **Results** |  |  |  |  |
| Participants | 20a | D;E | Describe the flow of participants through the study | Results: Dataset Characteristics |
|  | 20b | D;E | Report the characteristics overall and for each data source or setting | Results: Dataset Characteristics |
|  | 20c | E | For model evaluation, show a comparison with the development data | Results + Discussion |
| Model development | 21 | D;E | Specify the number of participants and outcome events in each analysis | Results & Appendix A |
| Model specification | 22 | D | Provide details of the full prediction model to allow predictions in new individuals | Appendix A |
| Model performance | 23a | D;E | Report performance estimates with confidence intervals, including for key subgroups | Results, though given it is a global bed prediction tool rather than a patient disposition model, subgroup analysis was not performed (see Methods) |
|  | 23b | D;E | If examined, report results of any heterogeneity in model performance across clusters | N/A (single centre) |
| Model updating | 24 | E | Report the results from any model updating (including the updated model and performance) | Results: Model Performance Across Training Cadences |
| **Discussion** |  |  |  |  |
| Interpretation | 25 | D;E | Give an overall interpretation of the main results, including issues of fairness | Discussion (whole) |
| Limitations | 26 | D;E | Discuss any limitations of the study and their effects on biases, uncertainty, and generalisability | Limitations |
| Usability of the model in current care | 27a | D | Describe how poor quality or unavailable input data should be assessed and handled | Methods: Data Source and Cohort (imputation strategy) |
|  | 27b | D | Specify whether users will be required to interact… and what level of expertise is required | Discussion & Conclusion |
|  | 27c | D;E | Discuss any next steps for future research, with a specific view to applicability and generalisability | Conclusion |

PROBAST + AI

**STEP 1**

| **Population** | Paediatric patients, age <16 years predominantly, with some overage patients up to 18 years, presenting to a tertiary paediatric emergency department. |
| --- | --- |
| **Index model** | Ensemble stacking model with mandatory XGBoost base learner + optional MLP, TabNet and logistic regression base learners; logistic regression meta-learner; BioClinicalBERT-derived embeddings from triage free-text; post-hoc probability correction. No retraining in base model. |
| **Comparator models** | Identical model architecture, though nine different retraining cadences were the comparisons to the static base model. |
| **Outcomes** | Inpatient admission from ED (to ward or emergency short stay ward) vs discharge home (including discharge against medical advice). Primary model outcomes are area under the receivor operator characteristic and absolute mean daily bed error. |
| **Timing** | Prediction is from the time of triage only. |
| **Setting and intended use of the prediction model** | Tertiary paediatric emergency department in Western Australia. It is intended for real time aggregated forecasting of daily inpatient bed demand from current ED arrivals to support live capacity planning and resource allocation. |

**STEP 2**

| **Classify the evaluation based on its aim** | | | |
| --- | --- | --- | --- |
| **Type of prediction study** | **PROBAST boxes to complete** | **Tick as appropriate** | **Definition for type of prediction model study** |
| Development only | Development |  | Prediction model development without external validation. These studies may include internal validation methods, such as bootstrapping and cross-validation techniques. |
| Development and validation | Development and validation | The publication contains both prediction model development and evaluation of performance on data not used for development. Specifically, temporal validation using rolling-window prospective simulation over 5 years. | Prediction model development combined with external validation in other participants in the same article. |
| Validation only | Validation |  | External validation of existing (previously developed) model in other participants. |

**STEP 3**

| **DOMAIN 1: Participants and data sources** | | | |
| --- | --- | --- | --- |
| **A. Quality** | | | |
| *Describe the sources of data and criteria for participant selection:*  Routinely collected real-world data from the only tertiary paediatric hospital in the state of Western Australia from July 1 2018 to June 30 2024, with all presentations included. | | | |
|  | | Dev | Val |
| 1.1 Were appropriate data sources used? | | Y | Y |
| 1.2 Was an appropriate study design used? | | Y | Y |
| 1.3 Did the in- and exclusions of study participants result in a representative dataset? | | Y | Y |
| **Risk of bias introduced by selection of participants** | **Quality concerns:**  *(low/ high/ unclear)* | Low | Low |
| *Rationale of quality rating:*  Only 477/409 784 excluded (technical incompleteness or rare non-admission/non-discharge outcomes). No exclusion based on predictor values or post-triage information. | | | |
| **B. Applicability** | | | |
| *Describe included participants, setting and dates:*  Broad range of data collecting, using routine triage data for paediatric patients to build a one year training and validation set then a temporally separate five-year simulation. | | | |
| **Concern that the included participants and setting do not match the review question** | **CONCERN:**  *(low/ high/ unclear)* | Low | Low |
| *Rationale of applicability rating:*  Relative to our research question, the applicability is optimal for a simulation study alone. However, addition of other sites is required for external validity. | | | |

| **DOMAIN 2: Predictors** | | | |
| --- | --- | --- | --- |
| **A. Quality** | | | |
| *List and describe predictors included in the final prediction model, how they were defined and assessed, and their timing of assessment:*  The predictors in the final ensemble stacking model include:   - Demographic features such as age (calculated from date of birth), sex, and postcode - Temporal features like arrival timestamp (with derived sine/cosine cycles, weekend indicator, and shift bins) - Triage-related elements including priority (Australasian Triage Scale 1–5) and chief complaint/code - Historical metrics such as days since last presentation/admission and count of presentations/admissions in the prior 12 months - Optional 768-dimensional NLP embeddings from triage description and nursing assessment free-text fields (generated via fine-tuned BioClinicalBERT with masked language modelling and contrastive fine-tuning).   These predictors were defined based on routine health record data, with missing values imputed appropriately (e.g., "unknown" for categorical, age based or zero for numeric), and assessed uniformly through triage nurse documentation, automated EHR queries, and computational derivation during preprocessing. All assessments occurred at the point of triage upon patient arrival, ensuring availability prior to clinician evaluation and alignment with the model's intended real-time use for bed-demand forecasting. | | | |
|  | | Dev | Val |
| 2.1 Were predictors defined and assessed in a similar way for all participants? | | Y | Y |
| 2.2 Was any pre-processing of predictors similar for all participants? | | Y | Y |
| 2.3 Were predictor assessments made without knowledge of outcome data? | | Y | Y |
| 2.4 Were the predictors included in the model available at the time the model was intended to be used? | | Y | Y |
| **Concern regarding the quality of the predictors or their assessment** | **RISK:**  *(low/ high/ unclear)* | Low | Low |
| *Rationale of quality rating:*  Met all criteria of this domain. | | | |
| **B. Applicability** | | | |
| **Concern that the definition, pre-processing, assessment, or timing of assessment of the predictors in the model do not match the review question or the assessor’s intended use** | **CONCERN:**  *(low/ high/ unclear)* | Low | Low |
| *Rationale of applicability rating:*  Used all data available and handling of data which simulates a real-world implementation considering all patients that present to the ED. | | | |

| **DOMAIN 3: Outcome** | | | |
| --- | --- | --- | --- |
| **A. Quality** | | | |
| *Describe the outcome, how it was defined and determined, and the time interval between predictor assessment and outcome determination:*  The outcome is binary inpatient admission from the paediatric emergency department, defined as transfer to an inpatient ward or emergency short-stay unit versus discharge home, including discharge against medical advice, to best reflect hospital bed demand. It was determined objectively from health records using the hospital’s disposition flag recorded at the conclusion of each patient's emergency department episode. | | | |
|  | | Dev | Val |
| 3.1 Were outcomes defined and assessed appropriately? | | Y | Y |
| 3.2 Were outcomes defined and assessed in a similar way for all participants? | | Y | Y |
| 3.3 Were outcome assessments made without use or knowledge of predictor data? | | Y | Y |
| 3.4 Was the time interval between predictor assessment and outcome assessment appropriate? | | Y | Y |
| **Concern regarding quality of the outcome or its determination** | **Concern:**  *(low/ high/ unclear)* | Low | Low |
| *Rationale of quality rating:*  All criteria met. | | | |
| **B. Applicability** | | | |
| ***At what time point was the outcome determined:***  Once a patient was discharged from ED (either to the ward or home)  ***If a composite outcome was used, describe the relative frequency/distribution of each contributing outcome:***  *N/A* | | | |
| **Concern that the outcome, its definition, assessment, or timing of assessment do not match the review question or the assessor’s intended use** | **CONCERN:**  *(low/ high/ unclear)* | Low | Low |
| *Rationale of applicability rating:*  Objective, hospital administrative measure which directly represents hospital bed capacity. | | | |

| **DOMAIN 4: Analysis** | | | |
| --- | --- | --- | --- |
| **A. Quality** | | | |
| *Describe the numbers of participants, number of candidate predictors, number of outcome events:*  The study included 409,307 paediatric emergency department presentations as participants after exclusions, drawn from a total of 409,784 over six years from July 1, 2018, to June 30, 2024. Candidate predictors numbered 19 structured features (demographics, temporal, triage-related, and historical) plus optional high-dimensional NLP embeddings (two 768-dimensional vectors from free-text fields), all considered for inclusion in the ensemble model based on validation performance. Outcome events consisted of 82,549 inpatient admissions (20.2% event rate), providing ample events per variable for robust model development and evaluation. | | | |
| *Describe how the prediction model was developed (e.g., with respect to modelling technique, predictor selection, and classification or risk group definition):*  The prediction model was developed as an ensemble stacking framework using mandatory XGBoost as a base learner alongside optional multilayer perceptron, TabNet, and logistic regression base learners, with a logistic regression meta-learner to blend outputs and incorporate structured triage features plus optional BioClinicalBERT-derived embeddings from free-text notes. Predictor selection involved including all predefined structured variables (demographics, temporal, triage-related, and historical) without elimination, while NLP embeddings were toggled (yes/no) during hyperparameter grid search on separate validation sets to minimise absolute mean daily bed error. For binary classification of inpatient admission versus discharge, class imbalance was addressed via Synthetic Minority Over-sampling Technique for XGBoost, followed by post-hoc probability recalibration based on training set distributions, with no explicit risk groups defined but rather continuous probability outputs for aggregated bed-demand forecasting. | | | |
| *Describe the performance measures of the prediction model, e.g., (re)calibration, discrimination, (re)classification, net benefit, and whether they were adjusted for optimism:*  The performance measures for the ensemble prediction model included discrimination via area under the receiver operating characteristic curve (AUROC, mean 0.843 for weekly retraining) and calibration via absolute mean daily bed error (AMDBE, mean 2.57), Brier score (mean 0.113), calibration slope (mean 1.041), intercept (mean 0.034), and expected calibration error with 10 bins (mean 0.031). Secondary classification measures encompassed accuracy (mean 0.846), sensitivity (mean 0.452), specificity (mean 0.947), precision (mean 0.686), negative predictive value (mean 0.871), and F1 score (mean 0.543), all reported with standard deviations across retraining cadences in a five-year temporal validation. These measures were adjusted for optimism through separate temporal validation sets, 5-fold cross-validation for the meta-learner, and hyperparameter optimization on non-training and non-testing data. | | | |
| *Describe missing data on predictors and outcomes as well as methods used for handling these missing data:*  Missing data on predictors were minimal, with postcode exhibiting the highest rate (n=3,093 out of 409,307 presentations), while age, sex, triage priority, and free-text fields had none; historical predictors like days since last presentation or admission were absent only for patients without prior records. These were handled through imputation: categorical variables used a "unknown" label, days since last presentation/admission were set to the patient's age in days if empty, and other numeric features were imputed as 0. Outcomes (inpatient admission) had no missing data among included presentations, as dispositions were fully recorded in electronic health records, with exclusions limited to rare non-binary cases (n=447). | | | |
|  | | Dev | Val |
| 4.1 Was there evidence that the sample size was reasonable? | | Y | Y |
| 4.2 Were continuous and categorical predictors handled appropriately? | | Y | Y |
| 4.3 Were participants with missing or censored data handled appropriately in the analysis? | | Y | Y |
| 4.4 If methods to address class imbalance were used, was the model or the model predictions recalibrated?* | | Y | Y |
| 4.5 Were methods used to address potential model overfitting? | | Y | Y |
| **Concern regarding quality of the analysis** | **Concerns:**  *(low/ high/ unclear)* | Low | Low |
| *Rationale of bias rating:*  All criteria met. | | | |
|  |  |  |  |

| **DOMAIN 4: Analysis** | | | | |  |
| --- | --- | --- | --- | --- | --- |
| **A. Risk of Bias** | | | | |  |
| *Describe the numbers of participants, number of candidate predictors, number of outcome events:*  The study included 409,307 paediatric emergency department presentations as participants after exclusions, drawn from a total of 409,784 over six years from July 1, 2018, to June 30, 2024. Candidate predictors numbered 19 structured features (demographics, temporal, triage-related, and historical) plus optional high-dimensional NLP embeddings (two 768-dimensional vectors from free-text fields), all considered for inclusion in the ensemble model based on validation performance. Outcome events consisted of 82,549 inpatient admissions (20.2% event rate), providing ample events per variable for robust model development and evaluation. | | | | |  |
| *Describe how the prediction model was developed (e.g., with respect to modelling technique, predictor selection, and classification or risk group definition):*  The prediction model was developed as an ensemble stacking framework using mandatory XGBoost as a base learner alongside optional multilayer perceptron, TabNet, and logistic regression base learners, with a logistic regression meta-learner to blend outputs and incorporate structured triage features plus optional BioClinicalBERT-derived embeddings from free-text notes. Predictor selection involved including all predefined structured variables (demographics, temporal, triage-related, and historical) without elimination, while NLP embeddings were toggled (yes/no) during hyperparameter grid search on separate validation sets to minimise absolute mean daily bed error. For binary classification of inpatient admission versus discharge, class imbalance was addressed via Synthetic Minority Over-sampling Technique for XGBoost, followed by post-hoc probability recalibration based on training set distributions, with no explicit risk groups defined but rather continuous probability outputs for aggregated bed-demand forecasting. | | | | |  |
| *Describe the performance measures of the prediction model, e.g., (re)calibration, discrimination, (re)classification, net benefit, and whether they were adjusted for optimism:*  The performance measures for the ensemble prediction model included discrimination via area under the receiver operating characteristic curve (AUROC, mean 0.843 for weekly retraining) and calibration via absolute mean daily bed error (AMDBE, mean 2.57), Brier score (mean 0.113), calibration slope (mean 1.041), intercept (mean 0.034), and expected calibration error with 10 bins (mean 0.031). Secondary classification measures encompassed accuracy (mean 0.846), sensitivity (mean 0.452), specificity (mean 0.947), precision (mean 0.686), negative predictive value (mean 0.871), and F1 score (mean 0.543), all reported with standard deviations across retraining cadences in a five-year temporal validation. These measures were adjusted for optimism through separate temporal validation sets, 5-fold cross-validation for the meta-learner, and hyperparameter optimization on non-training and non-testing data. | | | | |  |
| *Describe missing data on predictors and outcomes as well as methods used for handling these missing data:*  Missing data on predictors were minimal, with postcode exhibiting the highest rate (n=3,093 out of 409,307 presentations), while age, sex, triage priority, and free-text fields had none; historical predictors like days since last presentation or admission were absent only for patients without prior records. These were handled through imputation: categorical variables used a "unknown" label, days since last presentation/admission were set to the patient's age in days if empty, and other numeric features were imputed as 0. Outcomes (inpatient admission) had no missing data among included presentations, as dispositions were fully recorded in electronic health records, with exclusions limited to rare non-binary cases (n=447). | | | | |  |
|  | | A | I | E | |
| 4.1 Was model evaluation based on only apparent performance avoided? | | Y | | | |
| 4.2 Was there evidence that the sample size was reasonable? | | Y | Y | Y | |
| 4.3 Were participants with missing or censored data handled appropriately in the analysis? | | Y | Y | Y | |
| 4.4 If methods to address class imbalance were used, was the evaluation done in a dataset without imbalance correction?* | | Y | Y | Y | |
| 4.5 If data splitting was done to create training and test datasets, was there evidence that data leakage was avoided?* | |  | Y |  | |
| 4.6 If resampling methods were used to evaluate model performance, were all model development steps replicated in the resampling process?* | |  | Y |  | |
| 4.7 Was the predictive performance of the model evaluated appropriately, e.g., calibration, discrimination, and net benefit? | | Y | Y | Y | |
| **Risk of bias introduced by the analysis** | **RISK:**  *(low/ high/ unclear)* | Low | | | |
| Risk of bias for temporal external validation is low, however, it would be unclear for a different hospital setting, which is noted as a key limitation of this study. | | | | | |

**STEP 4**

| **Overall judgement about risk of bias and applicability of the prediction model evaluation** | |
| --- | --- |
| **Overall judgement of quality (development)** | **QUALITY CONCERN:** Low |
| *Summary of quality concern:*  Low concern for quality in model development, as all domains demonstrate rigorous participant selection, predictor assessment, outcome determination, and analytical methods with robust quality and minimal risk of bias. | |
| **Overall judgement of risk of bias (evaluation)** | **RISK OF BIAS:** Low |
| *Summary of applicability concerns:*  Low applicability concerns for model evaluation, as the temporal validation directly matches the specified PICOTS in terms of participants, predictors, and outcome within the intended tertiary paediatric emergency department context. | |

| **Overall judgement of applicability (development)** | **APPLICABILITY CONCERNS:** Low |
| --- | --- |
| *Summary of sources of potential bias:*  Low potential for bias in applicability during model development, as the study population, predictors, and outcome precisely match the defined PICOTS criteria without any unclear or high risk areas. | |
| **Overall judgement of applicability (evaluation)** | **APPLICABILITY CONCERNS:** Low |
| *Summary of applicability concerns:*  Meets all the criteria regarding the use of temporal validation as a form of external validation. The key limitation lies in the single site design which is a limitation of the study design. | |
